# Cognitive impairment appears progressive in the *mdx* mouse

**DOI:** 10.1016/j.nmd.2020.02.018

**Published:** 2020-05

**Authors:** Emine Bagdatlioglu, Paola Porcari, Elizabeth Greally, Andrew M. Blamire, Volker W. Straub

**Affiliations:** a John Walton Muscular Dystrophy Research Centre, Institute of Genetic Medicine, International Centre for Life, Newcastle University, Times Square, Newcastle upon Tyne NE1 3BZ, United Kingdom; b Institute of Cellular Medicine and Newcastle Magnetic Resonance Centre, Campus for Ageing and Vitality, Newcastle University, Newcastle upon Tyne, United Kingdom

**Keywords:** Duchenne muscular dystrophy (DMD), *Mdx* mouse, Magnetic resonance imaging (MRI), Cognitive behaviour

## Abstract

Duchenne muscular dystrophy (DMD) is an X-linked recessive muscle wasting disease caused by mutations in the *DMD* gene, which encodes the large cytoskeletal protein dystrophin. Approximately one-third of DMD patient's exhibit cognitive problems yet it is unknown if cognitive impairments worsen with age. The *mdx* mouse model is deficient in dystrophin demonstrates cognitive abnormalities, but no studies have investigated this longitudinally. We assessed the consequences of dystrophin deficiency on brain morphology and cognition in male *mdx* mice. We utilised non-invasive methods to monitor CNS pathology with an aim to identify changes longitudinally (between 4 and 18 months old) which could be used as outcome measures. MRI identified a total brain volume (TBV) increase in control mice with ageing (*p* < 0.05); but the *mdx* mice TBV increased significantly more (*p* < 0.01). Voxel-based morphometry (VBM) identified decreases in grey matter volume, particularly in the hippocampus of the *mdx* brain, most noticeable from 12 months onwards, as were enlarged lateral ventricles in *mdx* mice. The caudate putamen of older *mdx* mice showed increases in *T*_2_- relaxometry which may be considered as evidence of increased water content. Hippocampal spatial learning and memory was decreased in *mdx* mice, particularly long-term memory, which progressively worsened with age. The novel object recognition (NOR) task highlighted elevated anxiety-related behaviour in older *mdx* mice. Our studies suggest that dystrophin deficiency causes a progressive cognitive impairment in mice (compared to ageing control mice), becoming evident at late disease stages, and may explain why progressive CNS symptoms are not obvious in DMD patients.

## Highlights

• Longitudinal magnetic resonance imaging identified increased total brain volume in older *mdx* mice.
• Decreases in grey matter volume of *mdx* mouse hippocampus were noticeable from 12 months.
• *Mdx* mice demonstrated deficits in hippocampal long-term spatial learning and memory.
• Increased levels of anxiety-related behaviour shown by older *mdx* mice.

## 1 Introduction

Duchenne muscular dystrophy (DMD) is an X-linked recessive disease, occurring at an incidence of 1 in 3600–10,000 live male births [1,2]. DMD is characterised by a severe pathology of the skeletal musculature causing progressive loss of muscle, with premature death frequently occurring in the third decade of life as a result of cardiac and respiratory complications [3]. This fatal disease arises from mutations in the *DMD* gene; the largest gene in the human genome, with 79 exons spanning 2.4 Mb [4], and coding for a 427 kDa intracellular protein named dystrophin [5]. Although dystrophin is most abundantly expressed in muscle tissues, it is also expressed in the central nervous system (CNS) with the brain harbouring high amounts of various dystrophin isoforms [6,7]. Considerable research has been devoted to understanding the role of dystrophin in muscle cells; its role in the brain has received less attention until more recently.

Intellectual impairment has long been recognised as a disease symptom in DMD [8], and developmental delay has also been detailed by clinicians to be amongst the first signs at disease presentation. It is now generally acknowledged that the average IQ of DMD patients is 85, one standard deviation below the norm [9,10] and there is a higher incidence of neurodevelopmental disorders observed [11]. DMD patients display a varying degree of memory impairments, which have been attributed to the loss of dystrophin in brain structures involved in cognition, including the cerebellum and hippocampus, with approximately one-third of patients displaying some degree of cognitive deficit frequently manifesting itself in memory impairment, but ranging from reduced verbal intelligence [10,12] to severe autism [13,14]. Within the CNS full-length-dystrophin is highly expressed in pyramidal neurons of the hippocampus, which has important roles in memory formation and consolidation [6].

The *DMD* gene has 7 promoters resulting in various sized dystrophin protein products. The promoters located in the proximal region of the gene gives rise to three full-length dystrophin isoforms: Dp427b (brain) found predominantly in cortical neurons, Dp427m (muscle) and Dp427p (Purkinje) found to be expressed in mouse Purkinje cells but recently identified to be absent from the human cerebellum [15]. In additional to full-length dystrophin, there are five smaller dystrophin isoforms expressed in various body tissues. Dp140, Dp71, and Dp40 are also expressed in brain tissue, with cognitive impairment most prominent in patients with mutations in distal region of the *DMD* gene, which eliminates the expression of full-length dystrophin and one or more shorter dystrophin isoforms.

Many investigators have postulated roles for the dystrophin-glycoprotein complex (DGC) in the CNS, but unambiguous evidence has yet to emerge. Current literature suggests that neuronal DGC composition is complex, with numerous isoforms expressed in different tissues and neuron specific localisation [16,17]. It has been suggested that the DGC may be acting as an adaptor between the actin cytoskeleton and membrane bound receptors acting to anchor molecules which are critical for neuronal functioning [18]. The DGC may also participate in the formation and maintenance of macromolecular signalling complexes [19]. Furthermore, it is hypothesised that dystrophin may play a role in stabilising the postsynaptic apparatus during brain maturation in order to maintain a certain network status critical for synaptic plasticity [20]. Dp427, for example, has been found to colocalise with GABA_A_ receptors, and it is hypothesised that following GABA_A_ receptor insertion into the neural membrane, dystrophin is essential for their anchoring and clustering, which in turn is necessary for correct signal transduction. As a consequence, lateral diffusion of these receptors may be inhibited, hence contributing to their stabilisation [17,21]. Other dystrophin isoforms appear to be expressed in a cell-specific manner: Dp140 appears to be associated with microvascular glia cells [22], Dp116 is most abundantly found in Schwann cells of peripheral nerves, and Dp71 is the most highly expressed dystrophin in the brain (under control of the general promotor (G-dystrophin)) and is expressed in both neurons and glia [23].

There is conclusive evidence that the muscle pathology exhibited by DMD is progressive, and although previous studies have defined the cognitive deficit observed in DMD patients to be non-progressive [24], recent investigations are beginning to investigate cognitive impairment in older DMD patients (mean age 30.4 years old) compared to healthy controls [25]. Additionally, these previous studies do not correlate intellectual abilities with functional brain changes in the dystrophin deficient brain. Interestingly, recent research has identified progressive cognitive impairments in a small cohort of DMD patients. A significant decline in GABA_A_ receptors within the prefrontal cortices in DMD patients was identified with this observed decline more pronounced in older adult DMD patients (30–37 years old) [26]. Moreover, older DMD patients had an age-related decline in Wisconsin Card Sorting Test (WCST), a test predominately used to assess executive functions (strategic planning, organized searching, behavioural direction toward achieving a goal), compared to younger DMD adult patients (18–25 years old) which suggests that cognitive dysfunction does in fact progresses with age [26]. These studies demonstrate a progressive cognitive decline in older DMD patients, but these patients are still relatively young (within their 30′s) and suggests that cognition would deteriorate with increased age compared to healthy controls.

The most important mouse model for pre-clinical research in DMD is the *mdx* mouse. This model is routinely used for drug development, with the main focus upon treatment of muscle pathology. Previous studies in the *mdx* mouse have also shown deficits in passive avoidance learning [27], impairments in memory consolidation (both spatial and non-spatial learning) [28], and retention deficits at long delays in spontaneous alteration and bar pressing tasks [29]. One major caveat of the cognitive assessment testing in the *mdx* mouse is the lack of monitoring of potential progressive cognitive defects.

As therapy development for DMD has rapidly expanded in recent years, there is urgent need to develop reliable outcome measures to monitor disease progression and efficacy of treatment interventions. Successful clinical trials are reliant upon the validity of outcome measures, which are developed in the preclinical phase, to monitor the effect of a given intervention. Inaccurate preclinical work generating poor outcome measures hinders the utility of future clinical trials, as any observed effect may not truly be attributable to the intervention in question. It is important that standardised reproducible outcome measures are available to monitor cognitive dysfunction in the *mdx* mouse similar to those already available for assessing muscle pathology [30]. The limited number of sensitive outcome measures to assess and monitor the DMD brain phenotype, together with the need to avoid invasive techniques in patients makes magnetic resonance imaging (MRI) a particularly attractive option.

To date, almost all studies investigating cognitive dysfunction in DMD patients and in the *mdx* mice have detailed outcomes from cross-sectional studies using only a single age group. However, given that recent reports indicate that cognitive disability increases in DMD patients with increasing age [26], there is a need to assess how this disease parameter is effected by age. Consequently, the overall aim of this study was to apply already established techniques to non-invasively monitor cognition and brain function in *mdx* mice over time. Following a longitudinal assessment of a cohort of male *mdx* mice we found progressive changes in the brain morphology and cognitive abilities.

## 2 Materials and methods

The investigations conformed to the Guide for the Care and Use of Laboratory Animals published by the US National Institutes of Health (NIH Publication No.85-23, revised in 1985) and was performed under the terms of the Animals Scientific Procedures Act 1986, authorised by the Home Secretary, Home Office UK. All experiments were performed at the animal care facility of Newcastle University, UK. The study was approved by the Ethical Review Committee of Newcastle University. All animal experiments were performed under project licence number: PB3CA650C.

## 3 Animals

Male *mdx* (*C57BL/10*^ScSn-mdx/J^) mice were purchased from Jackson laboratories (Bar harbour, ME, USA) and male control (*C57BL/10*^ScSnOlaHsd^) mice were purchased from Harlan Laboratories (Indianapolis, USA). *Mdx* mice were bred with a homozygote male and a hemizygote female. This study utilised separate well established colonies of WT and *mdx* mice and did not use *mdx* mice littermate controls. Both genotypes are bred onto a *C57BL/10* background and are long-term colonies in our animal facility. Mice were group housed (4 mice per cage) under controlled temperature (17–28 °C) and light conditions (12:12 h light:dark cycle). Animals had free access to food and water.

### 3.1 Magnetic resonance imaging (MRI)

#### 3.1.1 Data acquisition

MR images were acquired using a 7.0 Tesla horizontal bore Varian scanner (Varian Medical System, Palo Alto, CA, USA) equipped with a 12-cm inner diameter actively shielded 37 gradient set (Magnex Scientific, Oxford, UK). A 39 mm (inner diameter) volume coil (Rapid Biomedical GmbH, Germany) was used as a radiofrequency (RF) transceiver for MR imaging of mouse brain.

Images were obtained following induction of anaesthesia with 4% Isoflurane (Abbott, USA) and 0.5 l/min oxygen in an anaesthetic chamber. For MRI brain examination, mice were laid prone on a home-made mouse holder keeping the head fixed by a bite bar in order to ensure that the head was positioned at the isocenter of the magnet. Body temperature was maintained using a warm air device (SA Instruments) set at 37 °C with monitoring via a rectal probe. Anaesthesia was maintained using 1.5% Isoflurane and 0.5 l/min oxygen via a nose cone controlled with regard to respiratory effort (∼50 bpm), monitored through an MRI-compatible small animal monitoring and gating system.

The MRI protocol included scout images to determine the correct positioning of the mouse brain, *T*_2_-weighted (*T*_2_-w) fast spin-echo (FSE) images and *T*_1_-weighted (*T*_1_-w) spin-echo (SE) images. For each mouse, axial, sagittal, and coronal *T*_2_-w images were acquired using the following parameters: repetition time/effective echo time (TR/TE_eff_) = 7000/40 ms, number of averages = 4, field of view (FOV) = 25 × 25 mm^2^, matrix (MTX) = 256 × 256, in plane resolution = 98 μm × 98 μm, slice thickness = 0.7 mm. Coronal *T*_1_-w SE images were acquired for each mouse using the following parameters: TR/TE = 900/16.07 ms, number of averages = 4, FOV = 25 × 25 mm^2^, MTX = 256 × 256, in plane resolution = 98 μm × 98 μm and slice thickness = 0.7 mm. The acquisition time for *T*_1_w and *T*_2_w imaging was 15 min per sequence.

*T*_2_ relaxation times of different brain regions (hippocampus, caudate putamen, cerebral cortex, corpus callosum, cerebellum) of control and *mdx* mice aged 6 and 18 months old were determined using a multi-echo-multi-slice (MEMS) sequence (TR/TE = 3500/8 ms, NE = 12, 18 slices, slice thickness = 1 mm, interslice gap = 0 mm in plane resolution = 196 × 196 µm^2^). For all FSE and SE protocols contiguous slices were obtained in an interleaved manner. *T*_2_-maps of mouse brain were computed for each slice on a voxel by voxel basis fitting the signal decay to the standard single exponential decay (with time constant *T*_2_) as a function of TE (12 echo times were employed, starting from 8 to 96 ms with an echo spacing of 8 ms). The acquisition time for the *T*_2_ mapping protocol was 15 min.

The animals spent on average a total of 90 min inside the MRI scanner per session.

#### 3.1.2 Brain volume estimations

Tissue volumes for whole brain and regional structures (hippocampus, cerebellum, ventricles) were estimated from the average values obtained from the *T*_2_-w scans utilising all orthogonal views. On average, 5 slices were used to calculate regional structural volumes. The cross sectional areas of each structure were determined by drawing regions of interest (ROIs) on each slice of the MRI images using ImageJ software (http://rsb.info.nih.gov/ij/). ROIs were outlined according to The Allen Mouse Brain Atlas (http://mouse.brain-map.org/). Total volume of each structure was then calculated for each mouse according to the following formula:

(1) $$\text{Total}\;\text{Brain}\;\text{Volume}\;=\;\Sigma_{j=1}^{n}d\cdot A_{j}$$

where **A_j_** is the cross sectional area on the ***j*th** slice through the mouse brain, **n** indicates the total number of the considered slices and **d** the slice thickness (0.7 mm).

All orthogonal views (sagittal, coronal, axial) and both *T*_1_*-*w and *T*_2_*-*w images were utilised for brain volume estimations.

#### 3.1.3 Voxel-based morphometry (VBM)

VBM describes the automated process of analysing morphological differences between images of the brain by performing voxel-wise statistics comparing groups of animals. VBM registers all mice into the same stereotaxic space accounting for differences in overall brain size and shape and then detects morphological changes regardless of changes in overall total brain volume (TBV).

*T*_2_-w MR images were processed using SPM12 software (Wellcome Department of Clinical Neurology, London; http://www.fil.ion.ucl.ac.uk). VBM was performed utilising the methods detailed by Sawiak et al. [31] and the freely available SPMMouse software package (http://www.spmmouse.org/) was used to provide the grey matter (GM) tissue probability map.

Following pre-processing, structural measures were modelled in a general linear model (GLM) to test hypotheses. An appropriate model for our study was a two-sample Student's *t*-test between the *mdx* and the control *C57BL/10* mouse brains. The images of this longitudinal brain study were acquired in-*vivo* with a relatively short scan time per mouse (15 min for a *T*_2_-weighted image) and as such the image quality was lower than the ex-vivo SPMMouse brain template used for the segmentations steps. With such a large number of tests, correction for multiple comparisons was necessary and we used the false-discovery rate (FDR) technique [32] with a threshold of *p* ≤ 0.05 for statistical significance.

The *mdx* mice developed kyphosis by the age of 18 months [33] and consequently this influenced how the mice lay in the MRI scanner. Due to this we had to perform a manual reorientation on all the *mdx* mouse brains in SPM12 prior to beginning with the SPMMouse segmentation (see *Supplementary Material* 1 for more detail).

For all MRI experiments 8 mice per genotype we repeatedly scanned between 4 and 18 months old in order to longitudinally assess anatomical brain changes in the same animal.

#### 3.1.4 Radiography

Mice were briefly anaesthetised with 5% Isoflurane in an anaesthetic chamber. Radiographs (X-rays) were performed on 18 month old mice with a Faxitron radiography system (Faxitron X-Ray, Lincolnshire, IL) at 23 kV for 5 s. DICOM raw images were then imported into ImageJ (https://imagej.nih.gov/ij/), and skull length measurements calculated by determining the width of the parietal plate and length of the nasal plate.

#### 3.1.5 Barnes maze testing

For all behavioural experiments mice were briefly handled by their tail in order to be placed into a small black tray used for transportation to the test arena. This approach was chosen to minimise handling and reduced stress.

We followed the same procedure as described by Sunyer et al. [34]. In brief, mice were given 3 min to explore the maze and locate the target hole (TH) associated with the target shape. In this time the primary errors (nose pokes not in the TH before it is found), total errors (number of errors committed before entering TH throughout the whole trial), primary latency (time to find the TH), and total latency (time to enter the TH) were recorded. Once inside the target box, located beneath the TH, the mice were left for 1 min before being returned to their home cages. There were 4 trials per day for 4 consecutive days, and in between each trial the mice were given a 15-min interval inside their home cage.

On day 5 (probe trial 1), the target box was removed. The TH for each mouse was the same as on previous days. The mice were allowed to explore the maze for 90 s and in that time a number of measures were recorded: primary latency, primary errors, total errors, and the preference for holes around the maze (total head pokes in individual holes around the maze). From the head pokes in various holes around the maze we created a success score. This was the number of head pokes in a hole, multiplied by a given value for that hole. Values were based on orientation around the target hole; with the target hole itself having a value of 10. The score for the individual holes was then totalled to create an overall success score. On day 12 (probe trial 2), the same procedure as on day 5 was performed with no training occurring between days 5 and 12.

We did not use any aversive stimuli. If mice failed to enter the TH during the allocated 3 min they were given a 1-min penalty in order to differentiate between the mice that entered in exactly 3 min and those that did not.

The behavioural tests commenced at the same time each day. As manual measurements were taken during the testing (e.g., number of errors) the investigator became a spatial cue and as such remained in the same position throughout the entire testing (i.e., across the training days and probe trials). The same investigator performed all behavioural testing and MRI scanning.

#### 3.1.6 The novel object recognition (NOR) task

Mice were given a 10-min habituation to an arena of dimensions 48 × 31 × 20 cm once a day for 4 consecutive days. On the fifth day, mice were exposed to the NOR task in two phases. In the first phase (sample), mice explored two identical objects (A1 and A2) for 3 min. Objects were either polystyrene spheres or stars, depending on randomisation. Objects were chosen based on difference in shape, to ensure novelty in the trials and also similarity in size to the mice. Mice were then given a 15-min inter-trial retention delay. Mice were then exposed to the second (choice) phase, in which the identical objects were replaced by one novel object (B1) and one familiar object (A3), and again mice explored these for 3 min.

Average time spent with the objects was recorded in both phases of testing. From this, D2 ratios’ were calculated for statistical analysis of the choice (test) phase. These create a value between +1 and −1, where +1 is total preference for the novel object, −1 is total preference for the familiar object, and 0 is no preference for either object. D2 ratios were calculated by determining the proportion of total time spent exploring either of the objects (B1-A3/B1+A3) [35,36].

For all behavioural assessments 8 mice per genotype between 4 and 12 months were examined. We excluded the 18 month old time point from behavioural investigations as it is argued that by this age *mdx* mice begin to demonstrate extensive muscle pathology which could impact mobility during both the Barnes maze and NOR tasks [37], [38], [39].

#### 3.1.7 Measurement of stereotypical behavioural

Stereotypical behaviour observed during behavioural testing was defined as a motor response which was repetitive, invariant, and seemingly without purpose or goal. Repetitive jumping, rearing, or circling the walls of the arena during the task were considered as a stereotypical behaviour unrelated to the behavioural tasks during this current study.

### 3.2 Statistics

All statistics were performed using IBM SPSS Statistics 22.0 software. A repeated measures ANOVA was used for multiple measurements taken from the same mouse over time (MRI longitudinal study, total/primary latency from Barnes maze testing) with students paired *t*-test used as post-hoc testing where a significant main effect was identified. A two-way ANOVA was used for *T*_2_ relaxation rates (6 and 18 months old), D2 ratio from the NOR task (4, 6, and 12 months old), and Barnes maze probe trials (at 4, 6, and 12 months). Bonferoni ad-hoc tests were used to determine where differences lay. A student's two-tailed *t*-test was used to compare control to *mdx* mice brain at 6 months and 12 months following VBM analysis. Level of significance was set at *p* < 0.05, or very significant *p* < 0.01. All graphs are presented as mean ± SEM.

### 3.3 Data availability

The data from this study is available upon request from the corresponding author (volker.straub@newcastle.ac.uk).

## 4 Results

### 4.1 Longitudinal non-invasive imaging

**Fig. 1:**
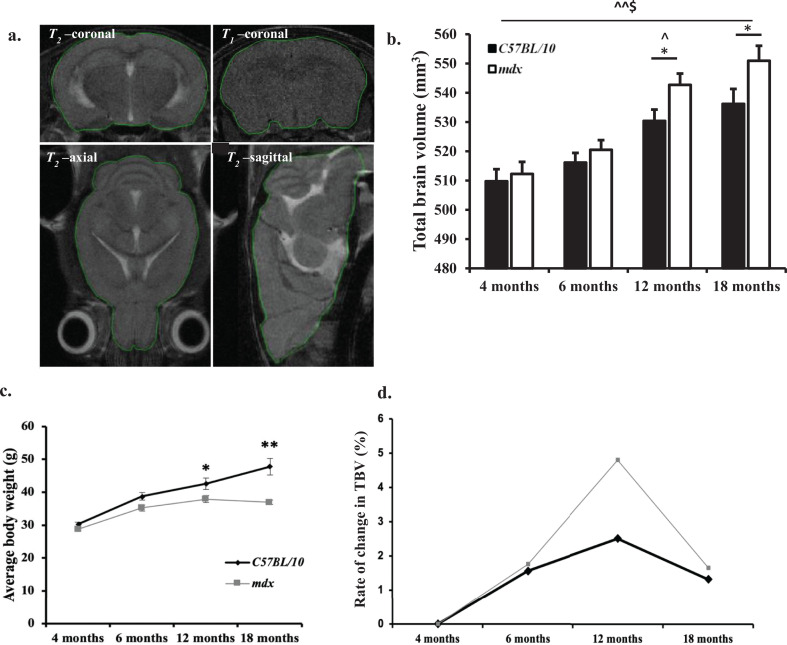
a–c: *Longitudinal total brain volume (TBV) measurements.* a**.** Representative images demonstrating the method used for estimation of total brain volume (TBV) in all mice using the polygon tool in ImageJ. The outline of each brain structure from representative *T*_1_-w coronal and *T*_2_-w -coronal, -axial and -sagittal images are delineated in green. b. Bar graph displaying longitudinal comparison of TBV. MRI derived TBV for mice at 4 months old, 6 months old, 12 months old and 18 months old (*n* = 8 mice per genotype at each time point). The control mouse brain increased in volume between 4 and 18 months old (*p* < 0.05). The *mdx* mice had a larger TBV compared to control mice from 12 months onwards (*p* < 0.01). Between 12 and 18 months old the *mdx* mice had a significantly larger TBV compared to control mice (*p* < 0.05). c**.** Line graph showing average body weights of mice used for the longitudinal MR imaging study. At 4 months old all mice have a comparable body weight. Between 12 and 18 months *mdx* mice begin to lose weight whereas control mice gain weight. Data presented as mean ± SEM, *p < 0.05, **p < 0.01 for control vs. *mdx,* ^*p* < 0.05, ^^*p* < 0.01 for changes observed with ageing in *mdx* mice, $<*p*.0.05 for changes observed with ageing in control mice. d. Line graph displaying the rate of change in TBV between 4 and 18 months old in control *C57BL/10* and *mdx* mice.

The objective of this study was to investigate structural and functional changes in the CNS of *mdx* mice over time. We first determined the total brain volume (TBV) of *mdx* mice using non-invasive imaging (Fig. 1a).

### 4.2 Enlarged TBV in *mdx* mice

Between 4 months and 18 months old control mouse brains increased by a total of 5.29 ± 1.03% with the largest increase in TBV observed between 6 months and 12 months old (3.05 ± 1.03%). The *mdx* mouse brain also increased in size between 4 months and 18 months old with an even larger TBV increase of 7.52 ± 1.38%, in contrast to the surge in increase of TBV observed in control mice, the *mdx* mice brains steadily increased in size between 4 months and 18 months old (Fig. 1b–d). Taken together, the results indicate that the control mouse TBV increases in size by a small but significant amount between 4 months and 18 months old (*p* < 0.05). However, the *mdx* mouse TBV increased considerably more (*p* < 0.01). There was a significant main effect between genotype*age on TBV (*F*_3,53_ = 26.22, *p* < 0.01).

### 4.3 Changes in lateral ventricle volume in the *mdx* mice

**Fig. 2:**
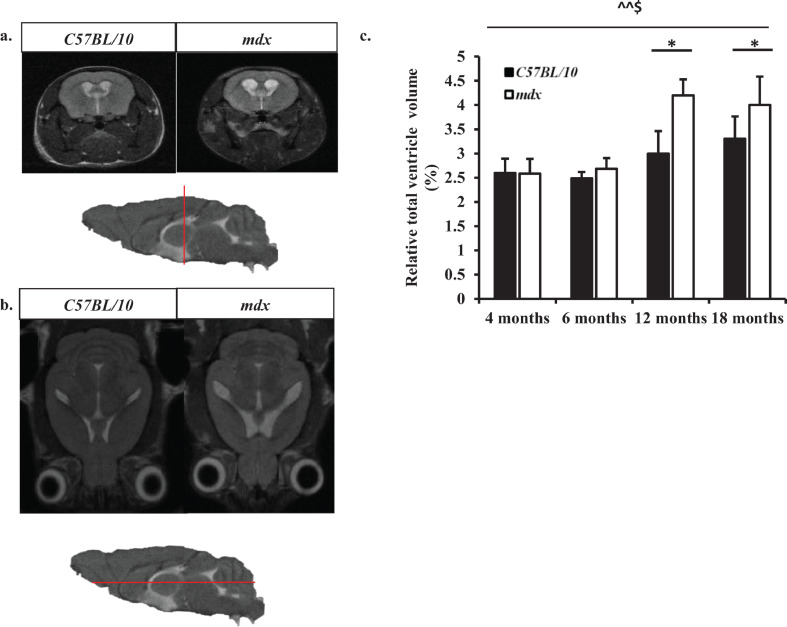
a–c: *Longitudinal ventricular volume measurements* a. Representative *T*_2_-w coronal MR images displaying increased lateral ventricles in *mdx* mice at 12 months old. Sagittal images where the red line indicates approximately where the coronal slice was acquired through the mouse brain. b. Representative *T*_2_-w axial MR images displaying increased lateral ventricle volume in *mdx* mice at 18 months old. Sagittal images where the red line indicates approximately where the axial slice was acquired through the mouse brain. c. Bar graph displaying the relative total ventricle volume between 4 and 18 months old. Relative total ventricle volume is calculated based on the total volume of the ventricle system expressed as a percentage of total brain volume. At 4 and 6 months old there was no difference between control and *mdx* total ventricle volume (*p* > 0.05). However, between 12 and 18 months old the *mdx* mice had a significantly higher total ventricle volume compared to age matched control mice (*p* < 0.05). Additionally, the relative total ventricle volume increased between 4 and 18 months old in both genotypes, but this increased volume was substantially larger in *mdx* mice (*p* < 0.01) compared to control mice (*p* < 0.05). Data presented as mean ± SEM, *p < 0.05, **p < 0.01 for control vs. *mdx*, ^*p* < 0.05, ^^*p* < 0.01 for changes observed with ageing in *mdx* mice, $<*p*.0.05 for changes observed with ageing in control mice.

**Fig. 3:**
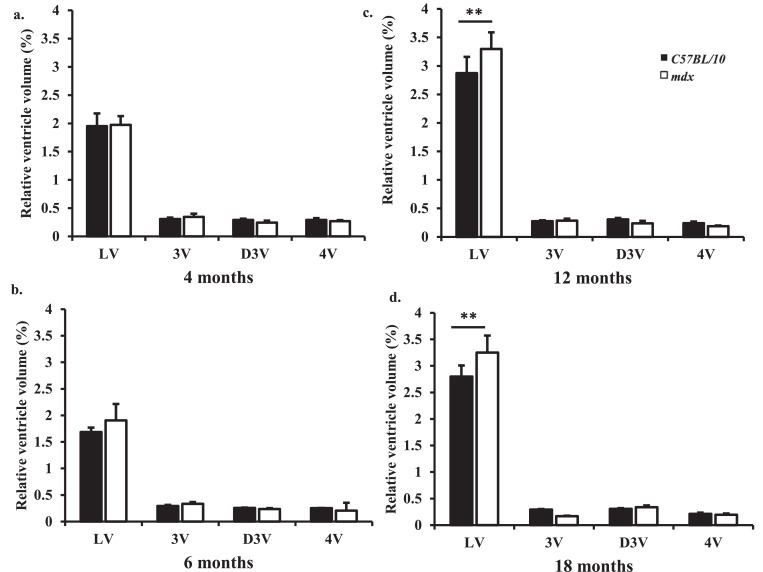
a–d: *Bar graphs displaying relative ventricle volume between 4 and 18 months old.* LV= lateral ventricles, 3V= third ventricle, D3V= dorsal third ventricle and 4V= fourth ventricle. a. Relative ventricle volume at 4 months old showing no difference in ventricle volume between control and *mdx* mice. b. Relative ventricle volume at 6 months old showing increased volume of the LVs in *mdx* mice but was not found to be significant (*P* = 0.06). c. Relative ventricle volume at 12 months old showing increased LV volume in *mdx* mice compared to aged matched control mice (*p*<0.01). d. Relative ventricle volume at 18 months old showing increased LV volume in *mdx* mice compared to aged matched control mice (*p* < 0.01). Data presented as mean ± SEM, *p < 0.05, ** *p* < 0.01, *n* = 8 mice per genotype at each time point.

The *mdx* mice brain ventricles were enlarged compared to age matched control mice (Fig. 2a,b). The dilation of lateral ventricles was particularly prominent in the coronal and axial MR images acquired and increased lateral ventricle volume was also observed (Fig. 2c), although this finding eluded statistical significance until 12 months old between control and *mdx* mice (*p*<0.01, control vs. *mdx* mice) (Fig. 3a–d). The ventricles were larger in mice aged 4 months old compared to mice aged 6 months old for both the control and *mdx* mice, suggesting that the brain volume increases faster than the brain ventricular system, given that these measurements are relative (Fig. 3a,b). There was a significant main effect between genotype*age on ventricle volume (*F*_3,29_=6.62, *p* = 0.01). Additionally, the *mdx* mice lost weight from 12 months onwards, whereas the control mice gained a significant amount of weight between 12 and 18 months old (Fig. 1c). There was a significant main effect between genotype*age on body weight (*F*_3,62_=32.72, *p*<0.01).

### 4.4 No difference in cerebellar volume between control and *mdx* mice

**Fig. 4:**
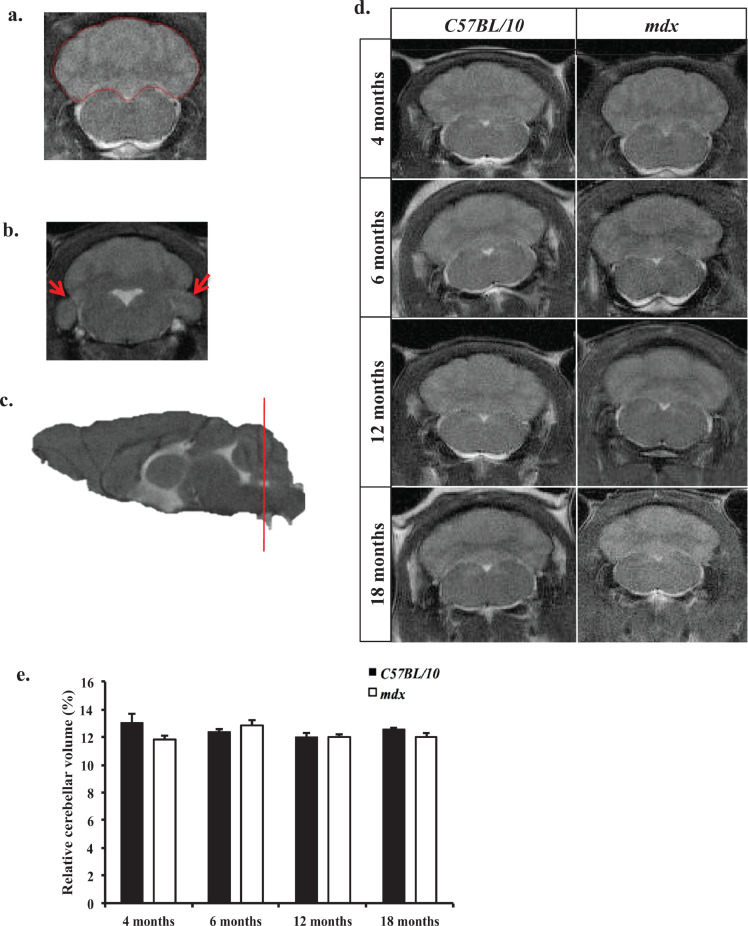
a–e: *Longitudinal cerebellar volume measurements***.** a. Representative coronal *T*_2_- w MR image of control mouse cerebellum delineated in red following ROI analysis in ImageJ. b. *T*_2_*-w* coronal MR image showing the paraflocculus and floculus lobules of the lateral cerebellum (red arrows) included in total cerebellar volume measurements. **c**. Sagittal *T*_2_-w MR image where red line indicates approximately where the coronal slice was acquired through the mouse brain. d. Representative *T*_2_*-*w images displaying the cerebellum from the same control and *mdx* mice between 6 and 18 months old. No gross anatomical differences were observed. e. Bar graph displaying relative cerebellar volume at all-time points investigated. We detected no changes in the relative cerebellar volume between control and *mdx* mice between 6 and 18 months old (*p* > 0.05). Relative cerebellar volume is calculated based on the total volume of the cerebellum expressed as a percentage of total brain volume. Data presented as mean ± SEM, *n* = 8 mice per genotype at each time point.

The cerebellum is a grey and white matter structure at the posterior end of the brain which has a distinctive web-like appearance seen in all three orthogonal views, the distinctive appearance is the result of the white matter cerebellar lobule strips appearing hypointense in a *T*_2_-weighted image. In the axial view, the cerebellum is located superior to the 4 V, the pons and the medulla (Fig. 1a). Fig. 4b shows the paraflocculus and flocculus lobules, with which the crus 1 ansiform lobule and the simple lobule make up the lateral cerebellar regions and have thus been included in all cerebellar volume measurements. There was no interaction between age*genotype on cerebellar volume (*F*_7,43_ = 2.84, *p* = 0.339).

The volume of the cerebellum was measured from serial coronal *T*_2_*-*weighted MR images of control and *mdx* mice, covering the same anatomical region in the brain and collected between 4 and 18 months old. We found no differences in the cerebellar volume (when expressed as a percentage of TBV) between control and *mdx* mice at any time point investigated (Fig. 4a–e).

### 4.5 No difference in hippocampal volume between control and *mdx* mice

**Fig. 5:**
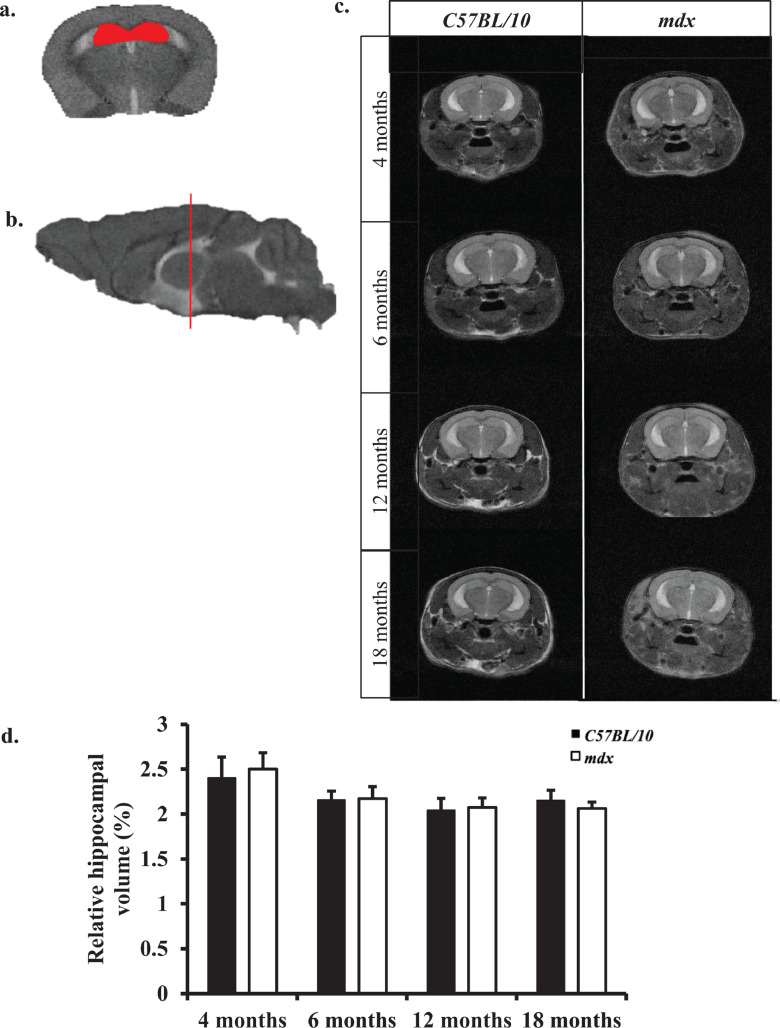
a–d: *Longitudinal hippocampal volume measurement.* a. Representative coronal *T*_2_*-*w MR image of control mouse hippocampus (red insert) following ROI analysis in ImageJ**.** b. Sagittal *T*_2_*-*w MR image where red line indicates approximately where the coronal slice was acquired through the mouse brain. c. Representative *T*_2_*-*w images displaying the hippocampus from the same control and *mdx* mice between 6 and 18 months old. No gross anatomical differences were observed. d. Bar graph displaying relative hippocampal volume at all-time points investigated. Relative hippocampal volume is calculated based on the total volume of the hippocampus expressed as a percentage of total brain volume. We detected no changes in the relative hippocampal volume between control and *mdx* mice between 6 and 18 months old (*p* > 0.05). Data presented as mean ± SEM following students *t*-test for control vs. *mdx* mice, *n* = 8 mice per genotype at each time point.

The volume of the hippocampus was measured using representative *T*_2_- weighted MR images from control and *mdx* mice covering the same anatomical region in the brain and collected between 4 and 18 months old. The whole hippocampus (CA1, CA3, DG and general hippocampal areas) was utilised for hippocampal volume estimations (Fig. 5a). Serial *T*_2_*-*weighted MR images were considered in order to accurately measure the entire volume of the hippocampus. Overall, we did not find any statistically significant difference in the volume of the hippocampus (when expressed as a percentage of TBV) between control and *mdx* mice at any time point investigated (Fig. 5a–d). There was no interaction between age*genotype on hippocampal volume (*p* = 0.14).

### 4.6 Changes in *T*_2_ relaxation rates detected in the *mdx* mouse brain at 18 months old

Quantitative MRI acquisition and analysis techniques allow investigations beyond conventional qualitative interpretation and provide further insights into brain pathologies. Relaxometry combines acquisition and analysis techniques to generate MR relaxation time constants that directly reflect the local environment of water. Since the transverse relaxation time, *T*_2_, is a quantitative measure of a basic biophysical property, which leads to signal contrast on MRI, it can provide a quantitative monitoring tool in both healthy and disease states. Increases in brain water *T*_2_ are associated with oedema (water content) and with changes to tissue microstructure.

**Fig. 6:**
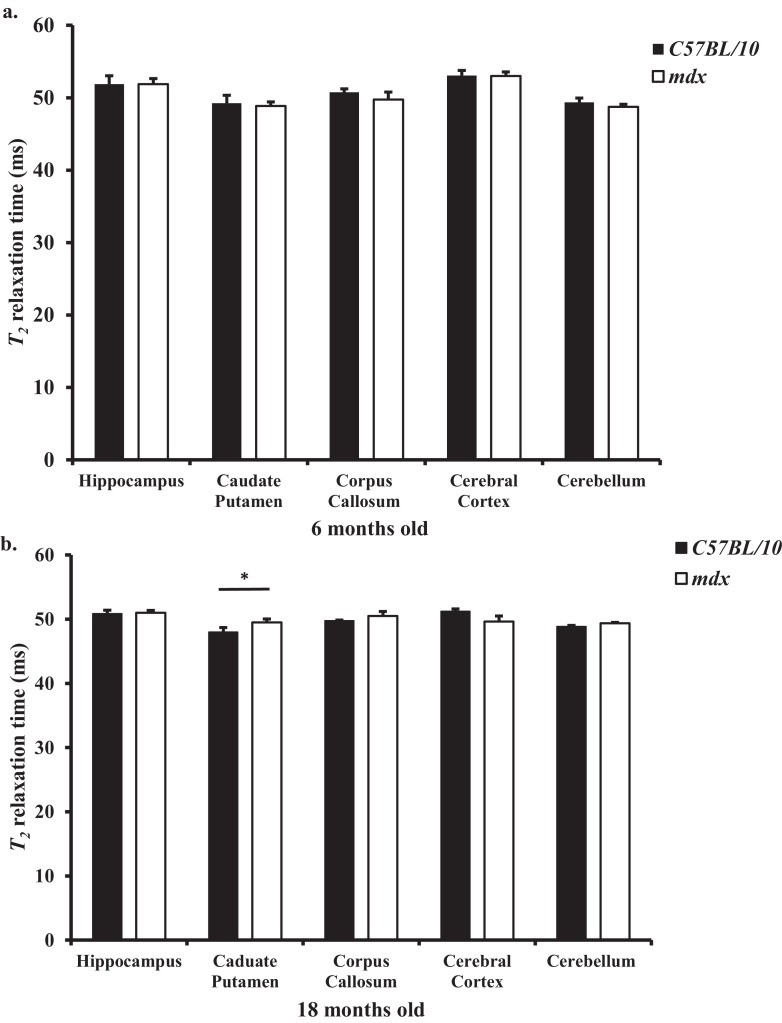
a,b: *Comparison of T_2_ relaxation times of each selected brain region between control and mdx mice.* a. At 6 months old there was no difference in the relaxation rate in any ROI between the control and *mdx* mice. b. At 18 months there was an elevated *T_2_* relaxation rate in the caudate putamen of *mdx* mice compared to aged matched control mice (*p* < 0.05). Data are presented as mean ± SEM, *p < 0.05, *n* = 4 mice per genotype at each time point.

We recorded the *T*_2_ relaxation times at 7T for numerous ROIs, including the cerebral cortex, corpus callosum, hippocampus, cerebellum, and caudate putamen, in *mdx* and control mouse brains at 6 and 18 months old (see *Supplementary Material* 2 for more detail). At 6 months old, no observation of change in *T*_2_ relaxation time was found in any ROI of either the control or *mdx* mouse brains (Fig. 6a) suggesting that the brain microstructure and water content between control and *mdx* mouse brain is comparable at this time point (*p* = 0.61). Interestingly, at 18 months old there was an elevated *T*_2_ relaxation rate within the caudate putamen of the *mdx* mouse brain (significant interaction between age*genotype for the caudate putamen (*F*_0.016,0.09_=4.61. *p* = 0.05) (Fig. 6b), therefore suggestive of vascular changes such as increased water content within this ROI.

### 4.7 Progressive changes in grey matter (GM) volume in the *mdx* brain

**Fig. 7:**
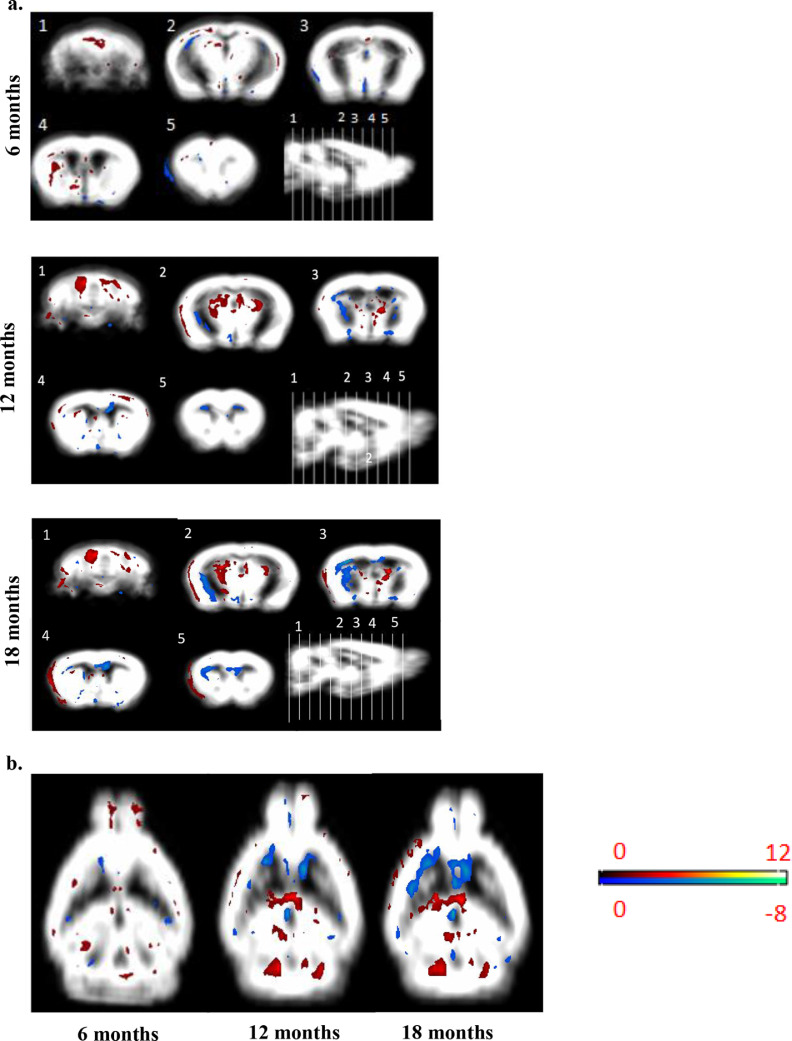
a,b: *Presentation of the VBM results produced by the SPM12 software with the SPMMouse plugin for grey matter of control versus mdx at various time points.* The grey matter average shown is for the control mice and a coloured overlay showing the location of significant clusters. Red clusters indicate where control mouse brain is larger than *mdx* brain and blue indicates where *mdx* brain is larger than control brain. Colour intensity on the scale bar refers to the level of significance with a lighter colour indicating a higher level of significance (*p* < 0.05, student's *t*-test). a. Coronal images numbered 1–5 with corresponding sagittal image detailed image plane. (Grey matter average is from 8 control mice). b. Axial grey matter slices detailing VBM findings.

VBM was applied to the *mdx* mouse brain between 6 and 18 months old. At 6 months old there was little difference in GM of control and *mdx* mice with changes observed mainly around the lateral ventricles and hippocampus, as well as the vermal cerebellum (Fig. 7a,b).

At 18 months old, VBM detected the largest number of differences between the control and *mdx* mouse brains. The GM was larger in control mice brains in numerous brain regions (following students paired *t*-test) including the thalamus, the inferior colliculus, the somatosensory cortex, and the hippocampus. In contrast, the *mdx* mouse brain had a larger GM volume than the control mice in various regions, mainly the cerebral cortex (lower layers), basal lateral amygdala nucleus, and the cingulate cortex (Fig. 7a,b).

### 4.8 Temporalis muscle hypertrophy in *mdx* mice

**Fig. 8:**
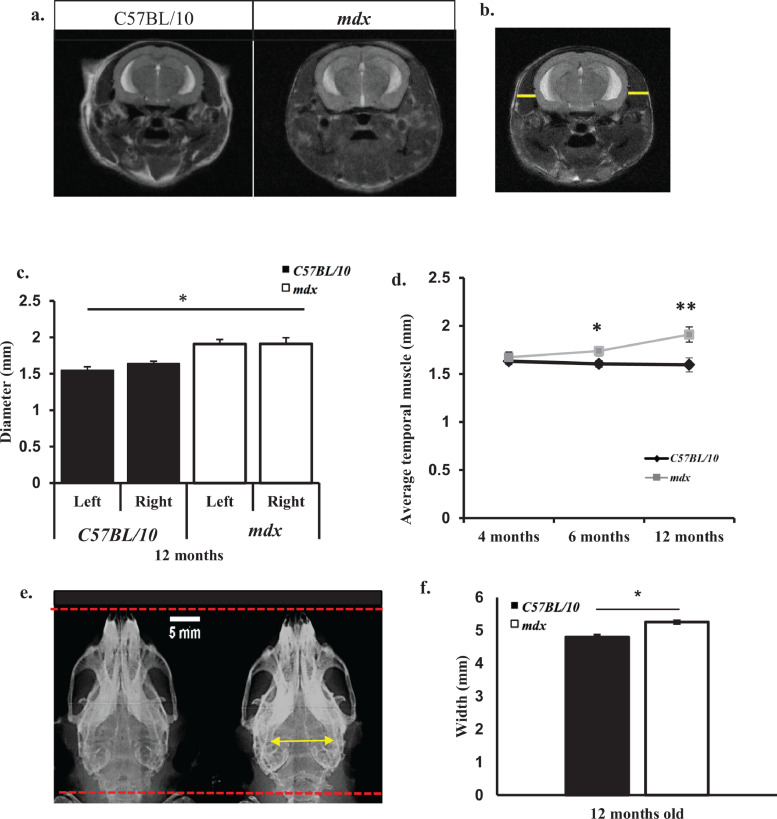
a–e: *Bilateral temporal muscle hypertrophy in mdx mice.* a. Representative *T_2_-*weighted coronal images showing changes in head shape of the *mdx* mouse. There is hypertrophy of the temporal muscle in *mdx* mice causing a change in head shape compared to age matched control mice. b. Representative image demonstrating temporal muscle thickness measurements (yellow lines). c. There is bilateral temporal muscle hypertrophy in *mdx* mice at 12 months old compared to aged matched control mice. d. Evolution of the temporal muscle thickness (averaged values for left and right) with age in control and *mdx* mice e. Representative radiographs of the mouse skull aged 12 months old (*n* = 4 mice per genotype). Yellow arrow represents widening of the squamosal bones. f. Bar graph displaying the widening of the squamosal bones in *mdx* mice at 12 months old compared to aged matched control mice (*p*<0.05). Data are presented as mean ± SEM, *p < 0.05, **p < 0.01 for control vs. *mdx* mice, *n* = 8 mice per genotype at each time point unless otherwise stated.

It has previously been demonstrated that the average temporal muscle thickness was significantly increased in DMD patients when compared to aged matched controls. In addition a rounder shape of the head compared to more ovoid shaped in healthy controls was also observed [40]. We found that the overall head shape of *mdx* mice differed to that of control mice, as indicated by temporal muscle hypertrophy observed qualitatively in Fig. 8a. We made measurements from both the right and left temporal muscle (Fig. 8b) and found bilateral temporal muscle widening in *mdx* mice between 6 and 12 months old (Fig. 8c,d), in addition to cranial changes (Fig. 8e). There appears to be changes in the head circumference, with *mdx* mice showing a larger, slightly rounder head shape at older ages (Fig. 8a–e). Radiographs also showed that *mdx* mice had a shortening of the nasal plate and widening of the parietal plate at 12 months old (Fig. 8e,f) contributing to a change in skull morphology. Additionally these findings are in congruence with previous studies, where no difference in skull morphology was identified in 3 month old mice [41], as it is only with increased age that we identified cranial changes.

### 4.9 Longitudinal cognitive profiling

To assess if cognitive impairment is progressive in *mdx* mice we performed two behavioural assays: the Barnes maze test and the novel object recognition (NOR) test, both of which rely upon intact hippocampal function [42] (see *Supplementary material* 3 for behavioural testing overview).

### 4.10 Barnes maze testing identified progressive deficits in the long-term memory of *mdx* mice

**Fig. 9:**
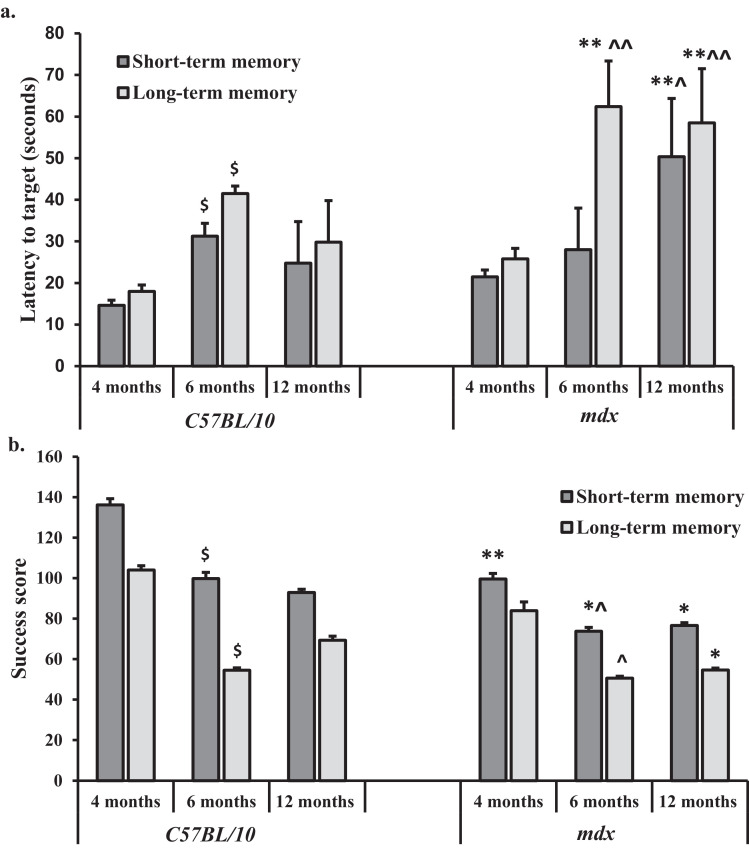
a,b: *Latency to find the target hole (TH) and the success scores during Barnes maze testing.* a. Short-term and long-term memory retention on the Barnes maze test. Short-term memory and long-term memory were assessed on day five and day twelve, respectively. A single trial was given to each mouse on the Barnes maze and the primary and total errors and latency(s) were evaluated as in the acquisition phase. b. Success score (hole value multiplied by the number of head pokes) observed during short and long-term memory retention trials. The highest success scores are observed during the short-term memory retention trials across all genotypes. The *mdx* mice had the lowest success score, at all-time points, for both short and long-term memory compared to aged matched control mice. Data presented as mean ± SEM, *p < 0.05, **p < 0.01 for control vs. *mdx*, ^*p* < 0.05, ^^*p* < 0.01 for changes observed with ageing in *mdx* mice, $<*p*.0.05 for changes observed with ageing in control mice.

The Barnes maze test was performed on separate cohorts of control and *mdx* mice aged 4–12 months old. A repeated measures ANOVA revealed that all mice showed significant spatial acquisition (learning) across all four days of testing at 4 months old (*F*_3,84_ = 20.9, *p* < 0.01), 6 months old (*F*_3,81_=6.62, *p* < 0.1) and 12 months old (*F*_3,84_=17, *p* < 0.05). The *mdx* mice had the longest mean latency to find the target hole (TH) for both short- and long-term memory between 4 and 12 months old. However, at 4 months old there was no significant difference between control and *mdx* mice with regard to short-term memory (*F*_3,28_ = 1.9, *p* = 0.1). However, at 12 months old the short-term memory of *mdx* mice was significantly impaired compared to at 4 months old where the mean latency was recorded as 50 ± 14 s and 21 ± 0.5 s, respectively. There was a significant interaction between genotype*age (*p* = 0.018). The long-term memory of *mdx* mice was also significantly impaired between 4 and 12 months old with a mean latency of 26 ± 2.5 s and 59 ± 13 s recorded, respectively. The control mice also showed a small decline in their short- and long-term memory abilities with a 10 s increase in the mean latency to target for both short and long-term memory between 4 and 12 months old (Fig. 9a). Additionally, the *mdx* mice made the largest number of errors, both for short-term and long-term memory probe trials, between 4 and 12 months old (see *Supplementary Material* 4 for more detail).

The *mdx* mice displayed a high level of anxiety related behaviour, expressed as a complete freezing response, with 2/8 *mdx* mice exhibiting this at 12 months old. Anxiety-related behaviour was not evident in the control mice at any time point investigated.

The highest success score was recorded during the short-term memory probe trial for both genotypes (Fig. 9a,b). The *mdx* mice had the lowest recorded success score for both short- and long-term memory at all-time points. The success score in the *mdx* mice decreased between 4 and 12 months old for both short- and long-term memory. At 4 months old, the *mdx* mice had a short-term memory success score of 99 ± 3 and 83 ± 1 for long-term memory. Whereas, at 12 months old the *mdx* mice had a short-term memory success score of 75 ± 1.5 and 53 ± 1 for long-term memory. In contrast, the control mice had a success score of 136 ± 3 for short-term memory and 105 ± 2 for long-term memory. Given that the success score decreased in *mdx* mice between 4 and 12 months old, these findings suggest that *mdx* mice have deterioration in cognitive function particularly in remembering the position of the target hole during long-term memory assessments.

### 4.11 The *mdx* mice showed a higher preference for the familiar rather than the novel object during the NOR task

**Fig. 10:**
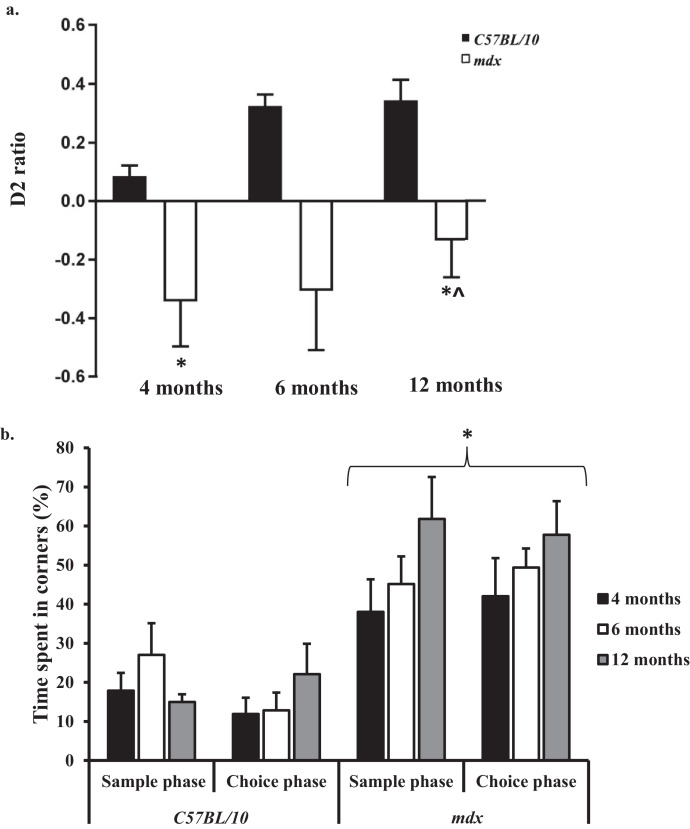
a,b: *Bar graphs displaying novel object recognition (NOR) task results.* a. Comparison of D2 ratios between 4 and 12 months old. The control mice had an increasing D2 ratio between 4 and 12 months old demonstrating an increased preference for the novel object. The *mdx* mice showed an increased preference for the familiar object at all-time points; however, the D2 ratio increased over time as the mice spent less time exploring the object in total. b. Bar graph displaying the amount of time that mice spent in the corners of the NOR arena between 4 and 12 months old. The control mice spent considerably less time in the corners during both the sample and choice phase compared to the *mdx* mice. At 4, 6 and 12 months old the *mdx* mice spent significantly longer in the corners during the sample and choice phases of NOR and this time increased with increasing age compared to control mice (*p* < 0.05). Data presented as mean ± SEM, *p < 0.05, **p < 0.01 for control vs. *mdx*, ^*p* < 0.05, ^^*p* < 0.01 for changes observed with ageing in *mdx* mice, $<*p*.0.05 for changes observed with ageing in control mice.

During the sample phase of the NOR task, both genotypes spent equal amounts of time with the two identical objects at all-time points (4–12 months old), demonstrating that both genotypes were equally interested with both objects (see *Supplementary Material* 5 for more detail). Overall, the *mdx* mice spent less time with the objects when compared to the control mice at all-time points investigated, suggesting that *mdx* mice have reduced exploratory behaviour possibly as a consequence of increased anxiety related behaviour (favouring to remain in the corners of the arena). During the choice phase of the NOR task at 4 months old, the *mdx* mice showed significant preference for the familiar object (11 ± 2 s) and spent significantly longer exploring the familiar object compared to the novel object (6 ± 1 s) (*p* < 0.05) (Fig. 10a), suggesting that the *mdx* mice may have a preference for the familiar and avoid the unfamiliar, which might be related to fear, although this is speculative. Additionally, during the choice phase of the NOR task at 6 and 12 months old the *mdx* mice also spent a longer amount of time investigating the familiar object compared to the novel object but they also spent a longer amount of time ‘frozen’ in the corners of the NOR arena (Fig. 10b). At all-time points investigated the control mice showed significant preference for the novel object, spending more time with the novel object with increasing age, represented as a higher D2 ratio (Fig. 10a) suggesting that the control mice have an intact recognition memory.

## 5 Discussion

### 5.1 Longitudinal non-invasive imaging

This was the first study to investigate longitudinal changes in the *mdx* mouse brain, scanning the same mice at each time point. A major finding of this study was that from 12 months onwards *mdx* mice has a significantly larger TBV compared to age matched controls. Previous MR imaging in *mdx* mice has not identified any differences in TBV; however, these studies utilised mice aged 3 months old, and our study too found no significant difference in TBV between control and *mdx* mice at 3 months [41]. It is only with advanced age that the *mdx* mouse TBV differed considerably to that of control mice. Similarly, our studies are in accordance with Miranda et al. who identified no change in TBV in the *mdx* mice at 6 months old. Additionally, the study also found there to be no significant differences in the volumes of the hippocampus or cerebellum in *mdx* mice (>6 months old) which is in agreement with the findings of this study [43].

A striking finding of the study was the increase in TBV in control mice, which was not an expected result. We observed significant, progressive, whole brain and regional volume increases (brain ventricle system) in mature control mice, where the increase began at 4 months old and brain growth slowed down from 12 months onward. In contrast, the human brain grows significantly between birth until our mid-teens, when brain growth begins to plateau, followed by a prolonged period of stability in brain size before a very slow decline (atrophy) in later life [44,45]. In rodents, the closure of the growth plates is long delayed, which is opposite to that in humans [46]. This effect, of increased TBV in rodents has only been documented once in a wild-type colony (C57BL/6 J background) [47], but is an important consideration when utilising control mice to assess changes in brain morphology.

The most recent study utilising MR imaging in DMD patients identified a reduction in TBV compared to that of healthy controls (although this was not found to be significant (*p* = 0.056)). Patients in this study were split into two groups, those expressing Dp140 (Dp140^+^) but not Dp427 and patients lacking Dp140 (Dp140^−^) and Dp427, with only the Dp140^−^ group showed a reduction in TBV. Additionally, most of the DMD patients in the study were currently taking steroids. Previous experiments have shown that steroids have an effect on brain morphology; hence authors could not rule out steroid use as a confounding factor for determining overall TBV. Utilising the *mdx*^4cv^ mouse (lacking Dp140 and Dp427) in future MR imaging experiments could help to determine how the loss of Dp140 affects the mouse TBV.

The lateral ventricles appeared to dilate significantly more in *mdx* mice, relative to TBV increases, suggesting that this is a pathological feature associated with full-length dystrophin deficiency. Furthermore, enlarged ventricles in both DMD patients and in the *mdx* mice have been previously reported at 10 months old. Within the choroid plexus (CP) basolateral membrane, utrophin A colocalises with dystrophin to control structural stability, transmembrane signalling, and ion/water homeostasis [48]. The loss of dystrophin could cause reduced stability of the ventricles, allowing increased CSF to circulate causing ventricular expansion. Moreover, several studies have described correlations between the degree of ventricular enlargement and the level of the cognitive dysfunction or mental retardation [49]. Although obstruction of the 4 V and/or the cerebral aqueduct has been known to cause ventricular expansion [50] this was not evident on any of the MR images acquired for the *mdx* mice. Additionally, enlargement of the ventricular system may also be caused by a failure of absorption or an overproduction of CSF, structural or functional impairments of cilia, and impaired cell proliferation around ventricles [51]. The ependymal cells of *mdx* mice were not investigated in this study, but further investigation is required to determine if dystrophin is necessary for the development of normal cerebral ventricles and neuroependymal integrity.

Recent reports identified an enlarged hippocampal volume in *mdx* mice aged 3 months old [52]; however, we did not find any statistically significant differences between the relative hippocampal volume of our control and *mdx* mice at any time point. Although at 18 months old the hippocampus was smaller in *mdx* mice this failed to reach statistical significance (*p* > 0.05) (Fig. 5d). This difference could be explained by the differing resolutions of the MR images obtained as our brain scanning was performed *in vivo* and hence our scan time per mouse was much shorter than the post mortem high resolution scanning performed on the 3 month old *mdx* mouse brains [41]. The higher resolution scanning allowed for better delineation of structures, in particular the hippocampus which borders are often hard to distinguish in lower resolution scans due to CSF in the ventricles obscuring the hippocampal boundaries.

Many researchers argue that the cognitive abnormalities associated with DMD could be classified as a cerebellar disorder due to the absence of full-length dystrophin (Dp427-p) in the cerebellum [53], and previous reports in the *mdx* mice detail increased density of dystrophin protein in the lateral versus the vermal mouse cerebellum [54]. We therefore included the entire cerebellum in our cerebellar volume measurements, but found no difference in the cerebellar volume or any gross structural abnormalities between control and *mdx* mice at any time point investigated, which is in accordance with studies in DMD patients [55,56]. These findings suggest that dystrophin loss does not cause any gross abnormalities in the cerebellum but may have a further impact at a cellular level, as previously reported in the *mdx* mouse [57]. Interestingly recently Dp427-p was found to be absent from the human brain [15] and it is evident that further studies are necessary to ascertain fully the role and expression of Dp427-p in both mouse and human cerebellum.

The characteristic pathophysiological conditions caused by blood-brain barrier (BBB) disruption are brain oedema resulting from an excessive increase of brain water content, inflammatory damage caused by infiltrating immune cells, and haemorrhage caused by the breakdown of microvessel structures. The *in vivo T*_2_ of brain tissue is related to the local water environment and free-to-bound water ratio, which may change in response to cellular and axonal loss or membrane breakdown. Changes in *T*_2_ relaxometry detected could signify a change in the brain water content of *mdx* mice at 18 months old in the caudate putamen only. The water content (*T*_2_ relaxation rate) of the *mdx* caudate putamen at 6 months old was comparable to that of the control mouse. These results suggest that older *mdx* mice may develop vasogenic cerebral oedema, as the BBB becomes disrupted due to the breakdown of tight endothelial junctions affecting the white matter through leakage from capillaries [58]. Dystrophin and other members of the DGC are expressed in brain microvessels [6], and in astrocyte perivascular endfeet [59] where dystrophin proteins play a role in BBB functions. Previous studies have identified that aquaporin-4 (AQP4) protein, the major water channel of the CNS, are associated with the DGC in the brain [60], at the BBB interface the *mdx* mouse brain shows an age-related reduction in AQP4 expression, with this reduction associated with swollen astrocyte processes [61]. AQP4 deficiency was also associated with BBB breakdown, causing vasogenic oedema and swollen perivascular astrocytes, indicating a close relationship between BBB integrity and control of the water flux by astroglial cells [60]. Our findings suggest, for the first time, the presence of vasogenic oedema *in vivo* in the *mdx* mouse brain, and that this pathological feature progresses with age, although further studies are necessary to confirm the presence of vasogenic oedema. One limitation of this study is that it is difficult to accurately delineate the rodent caudate putamen on the MRI images acquired as this structure does not have clear borders, therefore we chose to use a circular ROI placed into centre of the caudate putamen as we felt that this gave a good reflexion of the *T*_2_ values from this brain region.

Moreover, our findings highlight the need for ongoing dystrophin expression studies within the CNS. This study identified elevated *T*_2_-relaxation rates in the caudate putamen in the *mdx* mouse brain but there is little literature regarding dystrophin expression within this particular region. Additionally we also identified increased grey matter volume of the *mdx* caudate putamen (following VBM), which is in agreement with previous reports [41]. Unfortunately, the caudate nucleus and putamen are not distinguishable in rodents. The myelinated fibres that penetrate the striatum to connect to the cerebral cortex and subcortical structures do not form an internal capsule but are distributed throughout the striatum in the rodent brain. Thus, the rodent striatum is known as the ‘caudate putamen’. Functionally the striatum co-ordinates multiple aspects of cognition in both rodents [62] and humans [63]. Further studies are necessary to elucidate the role of the caudate putamen in the *mdx* mice, and further elucidate the expression pattern of dystrophin the brain by visualisation with in-situ hybridisation techniques.

VBM is routinely applied to the human brain to aid in the diagnosis of neurological disorders but its application in mouse brain is limited. VBM has been successfully applied to the study of other neurological disorders in mice [31], but this is the first study to apply VBM in the *mdx* mouse brain. We identified numerous changes with VBM in the *mdx* mouse brain where, the complexity of these changes increased between 6 and 18 months old. Interestingly, the *mdx* mice demonstrated enlargements in GM volume in the lower layers of the cortex, amygdala, and caudate putamen, in addition to reduced GM volume in the cerebellum, and hippocampus, regions known to be dystrophin rich, where the decrease in GM volume of these regions increased with age. These changes in the GM volume of the hippocampus and cerebellum could be linked to the cognitive dysfunction observed in *mdx* mice. The increased GM volume in the amygdala may be linked to the increased anxiety related behaviour, which we observed in older *mdx* mice. We believe that VBM is much more sensitive method for the detection of subtle changes in brain structure. The VBM findings complement the targeted and hypothesis led ROI analysis and allowed a data-driven analysis. Given that the main changes identified by VBM were in the hippocampus and cerebellum, structures which contain/are close to either high CSF or white matter in addition to grey matter, the boundaries on standard *T*_2_*-*w images for the hippocampus in particular could often be hard to distinguish manually due to CSF in the ventricles obscuring the view. We applied the minimum amount of post-processing to our VBM statistical data and present the analysis in the simplest form, with the result that the VBM data appears lightly noisy. We chose not to apply cluster-based thresholding approaches, which would improve the visual appearance by removing the smallest findings based on their physical extent. Future application of VBM approaches would benefit from the use of higher resolution image data, particularly with thinner slices than in our work, which would improve the intra-animal registration processes required for VBM.

It has been previously reported that thicker temporal muscles are associated with a more ovoid head shape in DMD patients compared to age matched controls [40]. We also observed a thickening of the temporal muscle in *mdx* mice and a rounder head shape, which is the first time these two disease characteristics have been reported by MRI in the *mdx* mouse. Taken together, this could suggest that the weaker, hypertrophic cranial muscles, including the temporal muscles, influence the development of the skull, resulting in a rounder shape. In DMD patients, it is shown that mastication is compromised and a decreased bite force is also reported. Given that the temporal muscle thickening increased in *mdx* mice between the ages of 4 and 18 months old, and their body weights decreased between these time points it is plausible that elevated muscle pathology in the temporal muscles causes mice to consume less food resulting in weight loss.

### 5.2 Longitudinal cognitive profiling

Unlike DMD patients, adult *mdx* mice do not show obvious progressive impairments in motor ability, presumably as a consequence of differences between mice and men regarding body size/architecture and a better regenerative muscle capacity in mice [64]. The Barnes maze test and the NOR task are not known to be strenuous activity tests, and thus were deemed acceptable behavioural assays to employ for the monitoring of a potential progressive cognitive dysfunction in *mdx* mice up to 12 months old. Moreover, the progressive cognitive abnormalities detected in a small cohort of DMD patients identified a progressive decline ultilising the WCST, which measures executive functions. The Barnes maze task is a goal-orientated task focusing on short/long-term reference memory (and hippocampal-dependent) but could also provide some information on executive functions in rodents including attention, inhibition, and working memory, given that the test involves the prefrontal cortex.

In this present study we utilised the Barnes maze test and numerous parameters (primary latency, total latency, primary errors, total errors, search pattern, and time spent in target quadrants) to monitor if cognitive impairment is progressive in *mdx* mice. It is only with a combination of these parameters that the progressive cognitive dysfunction becomes apparent. The ageing control mice showed a reduction in their success score (calculated during probe trials) between 4 and 12 months old with the largest reduction detected in long-term memory ability. The number of errors (primary and total errors) also increased with increasing age in control mice, but time spent in the target quadrant remained relatively constant between 4 and 12 months old during probe trials. These findings are consistent with previously published data regarding the effect of ageing on control mice (*C57BL6J*) behaviour [65], which suggested that impaired spatial and learning memory on the Barnes maze test is evident with increasing age, and further indicates that relatively narrow age differences can produce significant behavioural differences during adulthood in mice. In the *mdx* mice these parameters (success score, number of errors, time spent in the target quadrant) were affected to a greater extent. In particular, the time spent in the target quadrant, during short-term memory probe trial, was significantly reduced between 4 and 12 months old, as was the short-term memory (as shown by a decreasing success score and an increased number of errors). The long-term memory of *mdx* mice appears to show the highest deficit between 4 and 12 months old compared to the long-term memory defect of control mice.

Long-term memory impairment in DMD has recently been reported [28,66]. Dp427 appears to play a role in acquisition of associated learning as well as in general processes involved in memory consolidation following the assessment of *mdx* mice in this current study. Furthermore, it has been reported that long-term spatial memory is a function of the CA1 hippocampal sub-region and that this area has less of a role in short term memories [67]. Given that the Barnes maze test we performed highlighted in particular a reduction in long-term memory ability in the *mdx* mice between 4 and 12 months old, it is likely down to abnormalities in the CA1 field of the hippocampus.

The long-term memory impairments in *mdx* mice suggest a direct link with the loss of full-length dystrophin, therefore affecting all DMD patients. However, previous studies in the *mdx* identified that loss of full-length dystrophin has no effect on perception and gating of sensory input and does not impair spatial working memory performance (*mdx* mice aged 3–4 months old) [28]. Our study has highlighted that whilst at 4 months old spatial learning and memory in the *mdx* mice is comparable to that of control mice as the *mdx* mice age (6 months onwards), spatial learning and memory becomes significantly impaired.

Novelty and behaviour has gained much attention and interest from researchers. Novelty can be defined as an alteration from the expected likelihood of an event on the basis of both previous information and internal estimates of conditioned probabilities [68]. A novel stimulus can affect an animals’ behaviour and the NOR task relies on the rodents’ innate exploratory behaviour in the absence of externally applied rules or reinforcement [35,36]. The preference for the novel object means that presentation of the familiar object exists in the animals’ memory [68]. The recognition of novelty requires more cognitive skills from the subject, relative to tasks measuring exploration of novel environments

It is widely acknowledged that NOR paradigms are influenced by both hippocampal and cortical lesions [42]. The hippocampus is important for object recognition memory; damage to hippocampal structures causes moderate and reliable anterograde memory impairment. A rodent that remembers the familiar object will spend more time exploring the novel object [35]. DMD patients have demonstrated impairments in visual recognition memory (during Peabody picture vocabulary tests) [69]. The *mdx* mice displayed a preference for the familiar object, and were opposed to the novel object, at all-time points, which may rather suggest that *mdx* mice avoid the novel object because of anxiety and prefer to stay with the familiar one. As *mdx* mice didn't spend the same amount of time with both objects, which would suggest impaired visual memory, the NOR task may measure more than visual memory. The time spent with the familiar object did not increase over time but the amount of time that the mice spent with the objects in total decreased between 4 and 12 months old. These findings highly suggest that the loss of Dp427 from the *mdx* hippocampus has a direct effect on recognition memory formation, given the higher preference for the familiar object the *mdx* mice exhibited. The amount of time the *mdx* mice spent in the corners (frozen) of the NOR arena increased between 4 and 12 months old and is indicative of increased anxiety-related behaviour. This freezing behaviour has previously been reported in *mdx* mice, but this is the first time this anxiety-like behaviour has been observed to increase with increasing age in *mdx* mice.

Remarkably *mdx* mice also displayed repetitive behaviour, consistent with autistic-like traits in mice [70]. At 12 months old 3/8 *mdx* mice displayed stereotypical behaviours associated with autistic traits in mice including spontaneous motor stereotypes: circling the inside walls of the arena and jumping repeatedly. These characteristics were most prominent in *mdx* mice at 12 months old. Additionally, *mdx* mice displayed similar traits to those seen in mice of neurodegenerative disease. For example the earliest cognitive deficits in mouse models of Alzheimer's disease have been shown at 6 months of age by multiple groups [71,72], and Alzheimer's mice showed reduced spatial learning and memory during the Barnes maze task [73].

The control mice showed an increased preference for the novel object with increasing age and these findings are in congruence with previous literature examining the effects of ageing on rodents’ performance during the NOR tasks [74].

Furthermore, previous reports detail a potent freezing response in male *mdx* mice, expressed as lasting tonic immobility, reminiscent of the natural defensive death-feigning posture that animals exhibit when confronted by a predator or other threat, which is theorised to involve a brain circuit that includes the amygdala [75]. Authors suggest that if *mdx* mice were group housed rather than singly housed, and exposed to a familiar environment, this response could be reduced. In this present study male *mdx* mice were group houses (average 4 males per cage) and exposed to the same maze for 5 consecutive days, and yet a proportion of *mdx* mice still exhibit complete lasting tonic immobility (freezing response) at 12 months old during the Barnes maze test. This phenotype could be used to potentially assess the dynamics of therapeutic agents targeting brain dysfunctions in this model of DMD, as similarly suggested by Vaillend and Chaussenot [75].

Although *mdx* mice were subjected to repeated behavioural testing, the testing sessions were very short (<15 min total per day, with an inter trial interval of 15 min) and were only performed for 5 consecutive days. Neither the NOR task or the Barnes maze test are strenuous activities, therefore, we do not believe that fatigue related to muscle structure and/or functional integrity had any impact on how the *mdx* mice performed the task. Additionally, other studies demonstrate that muscle impairments are not noticeable in the *mdx* mouse model until much older in age, hence we did not include any 18 month old *mdx* mice in our behavioural assessments [37], [38], [39].

## 6 Summary

Overall, our study suggests that there is a progressive decline in cognitive function with age in *mdx* mice. Utilising a combination of techniques this is the first study, to the best of our knowledge, to demonstrate clear progressive CNS pathology in the *mdx* mice. We have identified non-invasive measures to detect several longitudinal changes in cognitive function and brain morphology in the *mdx* mouse, including: enlarged cerebral ventricles; larger total brain volume; and increased primary latency on the Barnes maze test. These findings have important implications for future studies examining the neurophysiological consequence of neuronal dystrophin deficiency and how its loss impacts brain morphology and cognition overtime. As the life expectancy of DMD patients has increased considerably over the past few decades [76], a better understanding of the cognitive profile in older DMD patients is vital as a progressive cognitive impairment will have direct implications on DMD patients’ overall quality of life.

This study suggests that dystrophin-deficiency results in late onset neurodegeneration that isn't observed in DMD patients because of their reduced life expectancy. Becker muscular dystrophy (BMD) patients on the other hand still have residual dystrophin expression and therefore might not show the same late-onset progression of CNS symptoms as observed during this *mdx* mouse study.

## Appendix Supplementary materials

Image, application 1
