# Supplementary material for: Cognitive impairment appears progressive in the mdx mouse
Source: Neuromuscul Disord. 2020 May;30(5):368–88. doi: 10.1016/j.nmd.2020.02.018 (PMC7306157; doi:10.1016/j.nmd.2020.02.018)
Supplement: Supplementary file 1 [file mmc1.docx]

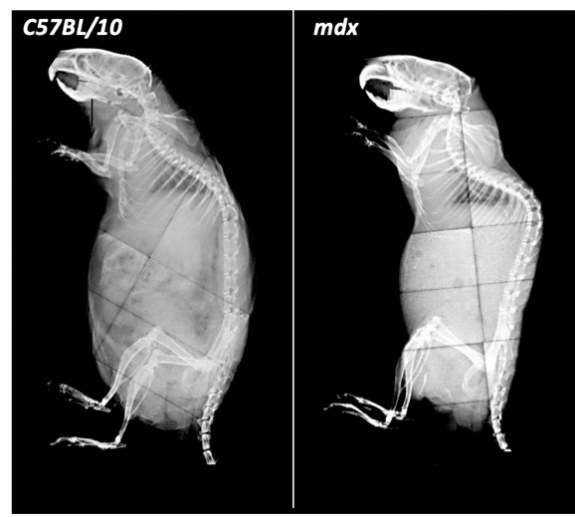


**a.**

B

C

D

A

KI = AB/CD


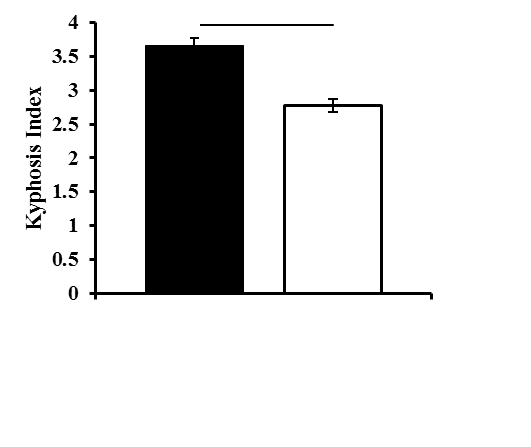

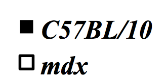


******

**b.**


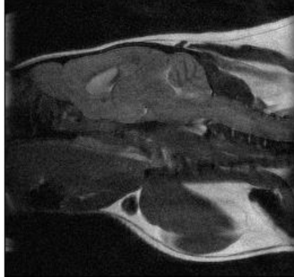

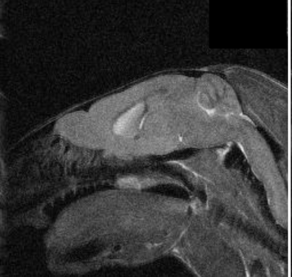


***C57BL/10***

***mdx***

**d.**

**c.**

**18 months**

**Supplementary material 1**

**Impact of Kyphosis during MRI scanning.** **a.** Examples of mouse whole body radiographs used for calculation of (Kyphotic Index) KI at 18 months old. The spinal deformity results in a decrease in KI and alteration of thoracic shape and size. **b.** KI in mice calculated from radiographs of anesthetised mice positioned in right lateral recumbency. Line AB is the length of a line drawn from posterior edge of C7 to the posterior edge of L6, usually where it contacts the wing of the ilium (which is more consistently identifiable than the sacral border). Line CD is the distance from line AB to the dorsal border of the vertebral body farthest from that line. KI = AB/CD. **c.** KI of 18 months old control and *mdx* mice. Mice were radiographed at 18 months old and measurements were averaged. Values are mean ± SEM. n=4 mice/genotype, ** p<0.01 following students t-test. **d.** Representative *T_2_*-weighted sagittal images demonstrating the downward angulation of the *mdx* brain in the MRI scanner at 18 months old due to increased levels of kyphosis. Consequently, the brains from the *mdx* mice had to be manually reoriented before being processed in the SPMMouse software for VBM analysis.


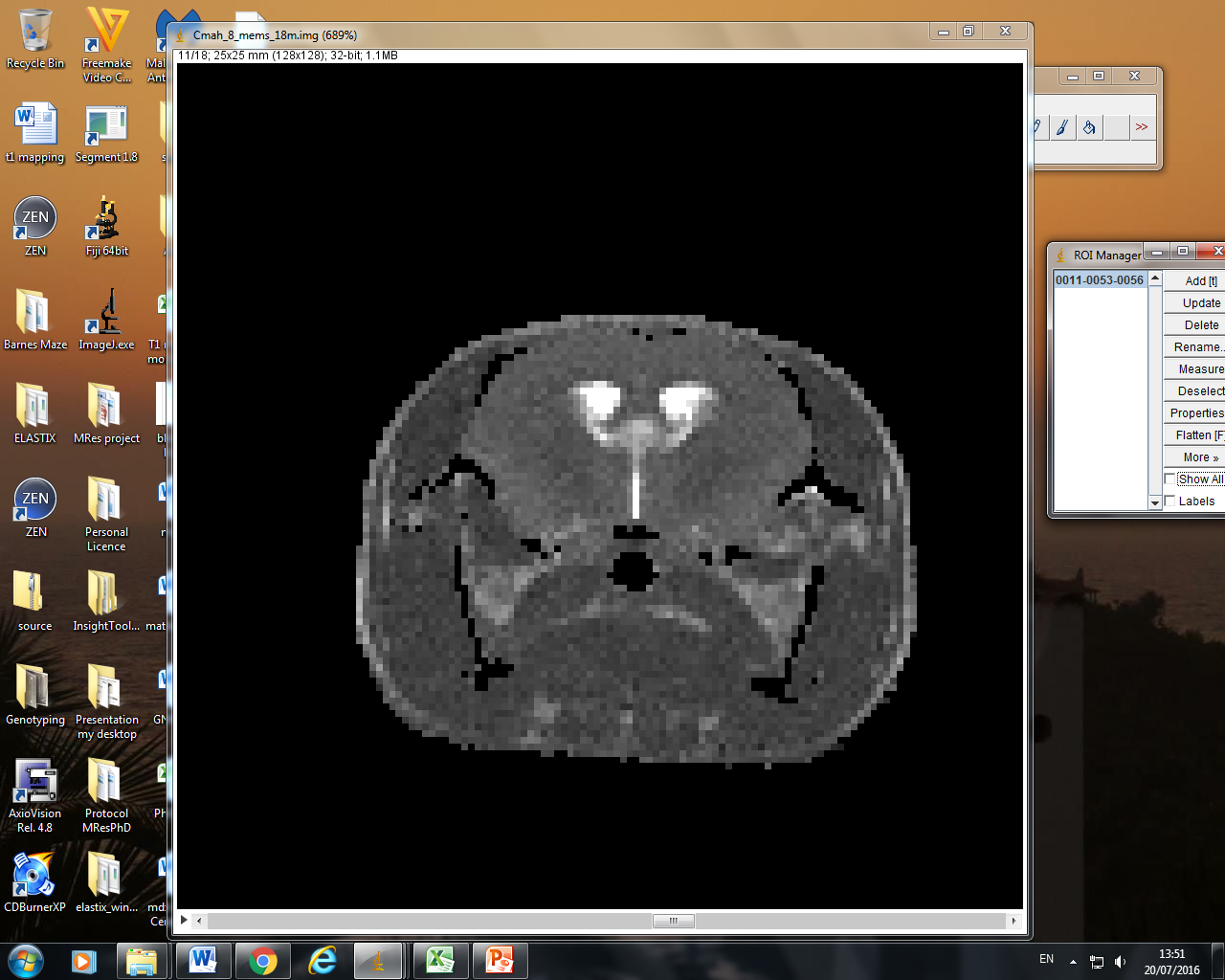

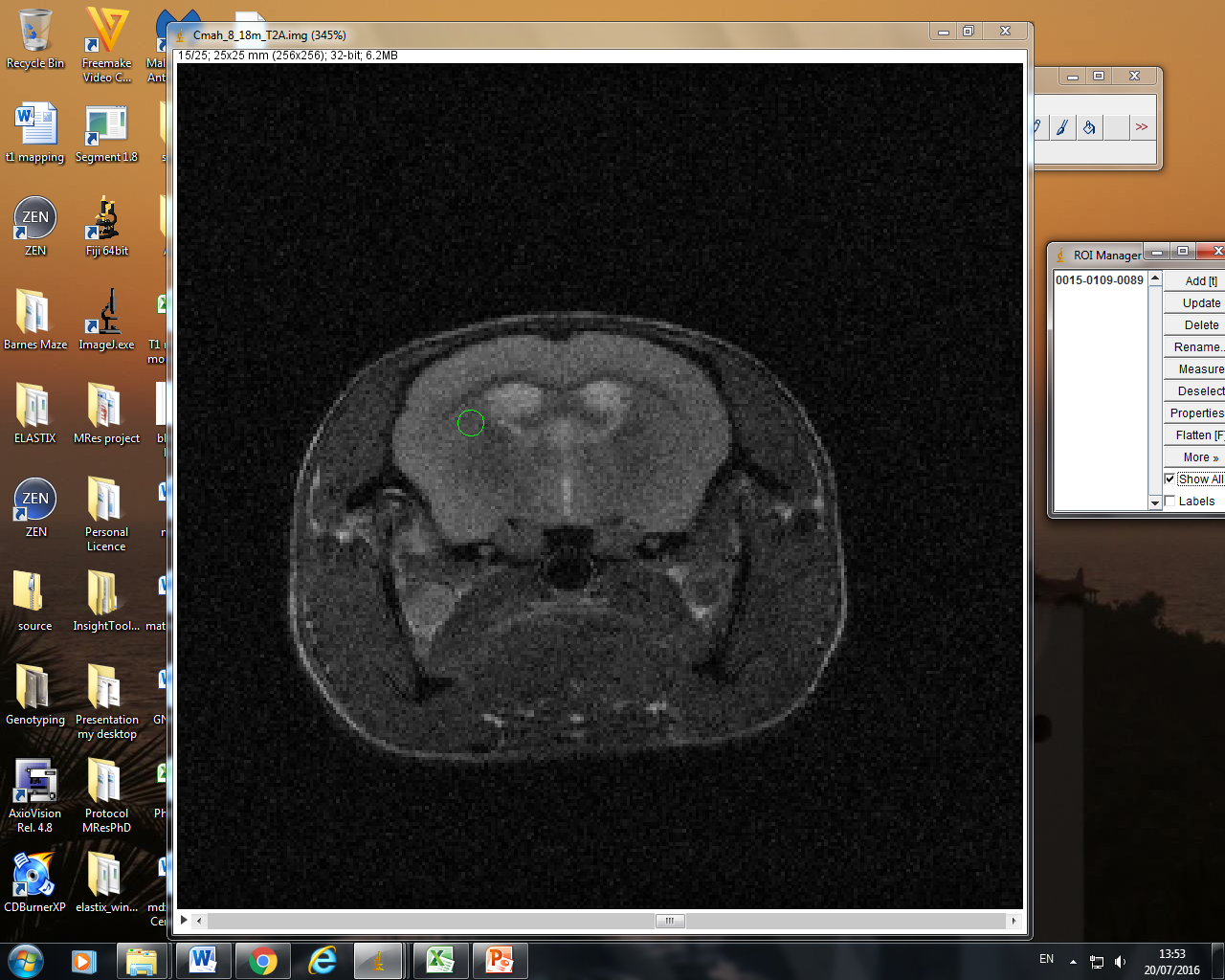

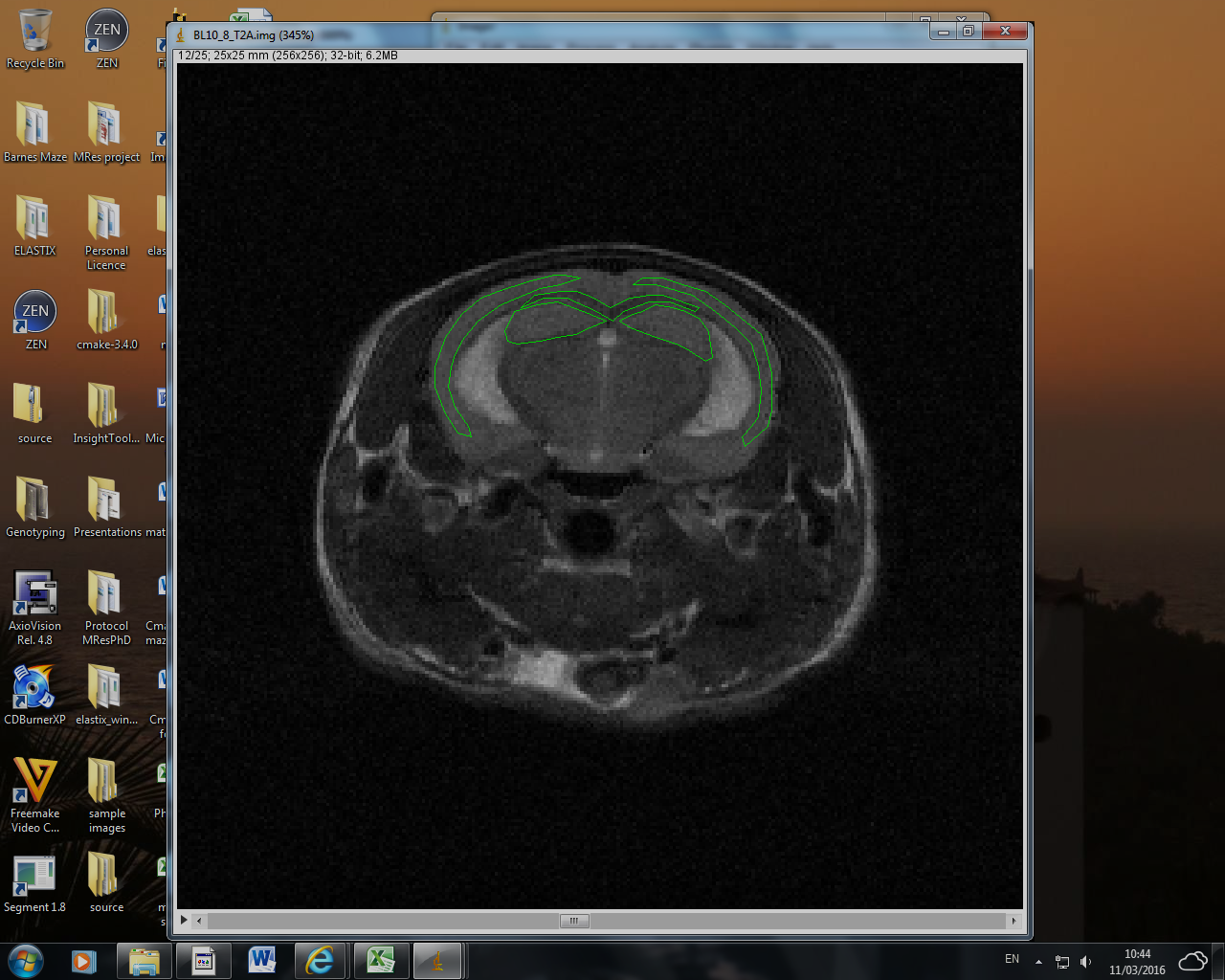


ROI_1

ROI_2

ROI_2

ROI_3

ROI_1


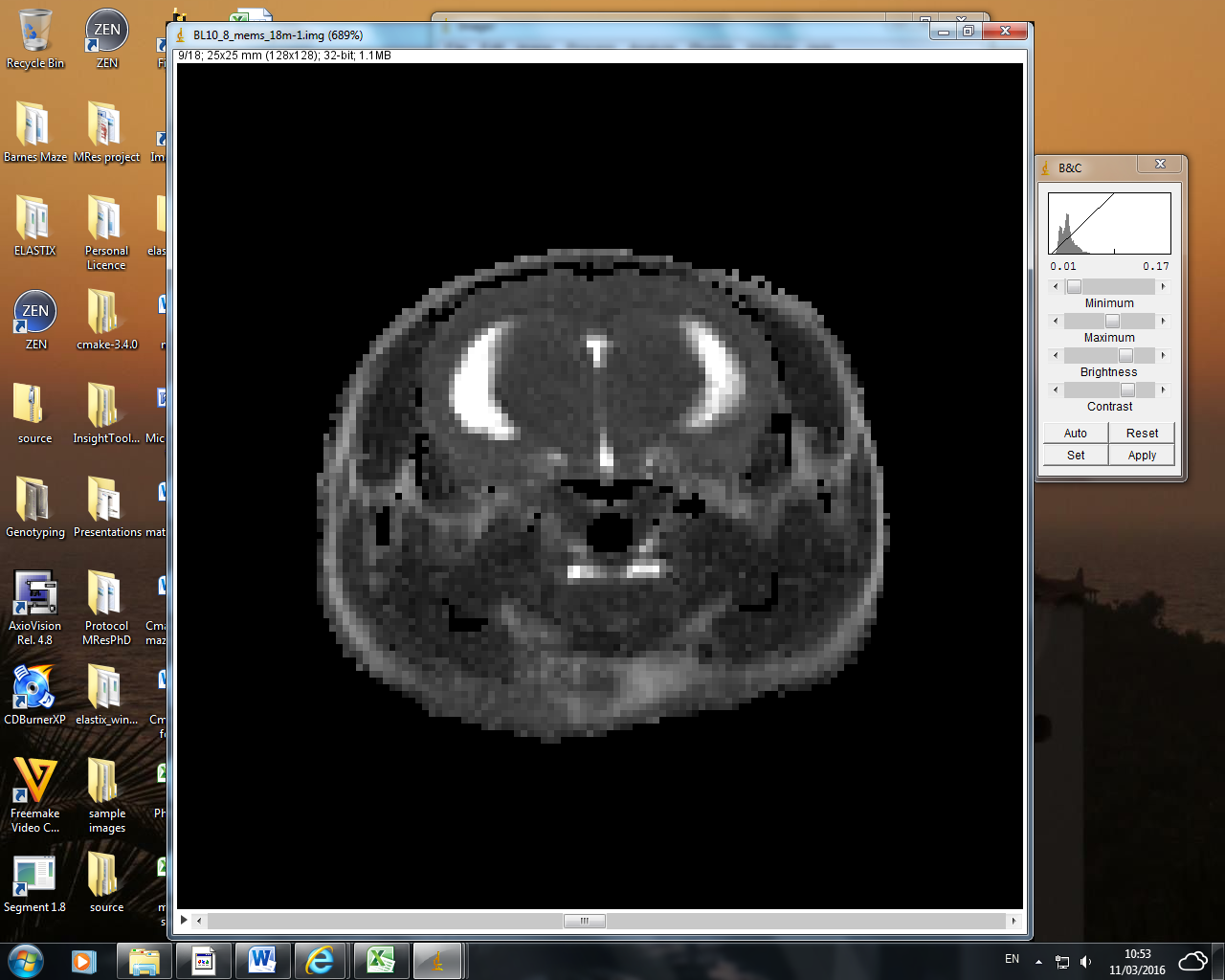


**a.**

**c.**

**ROI_4**

**b.**

**d.**


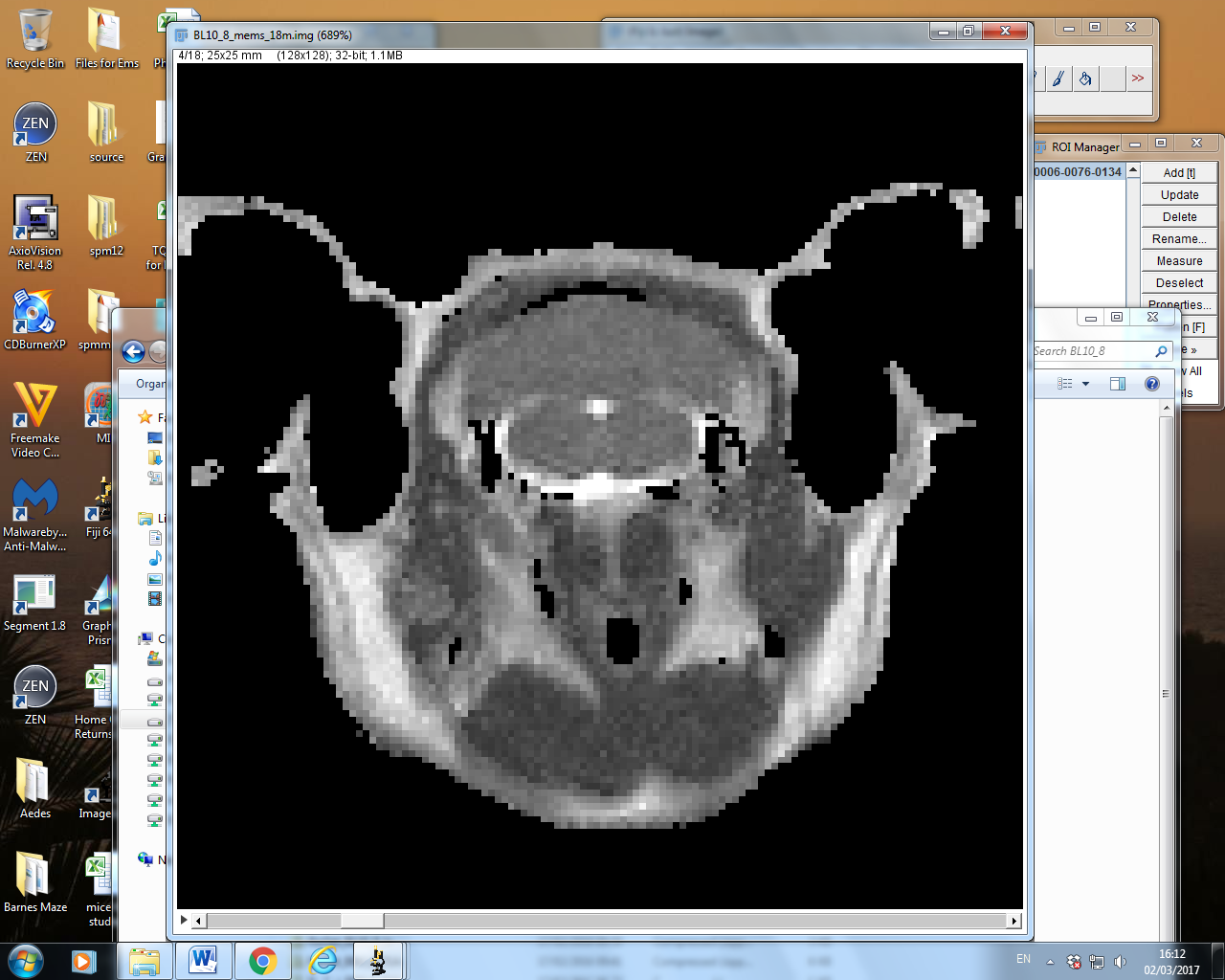

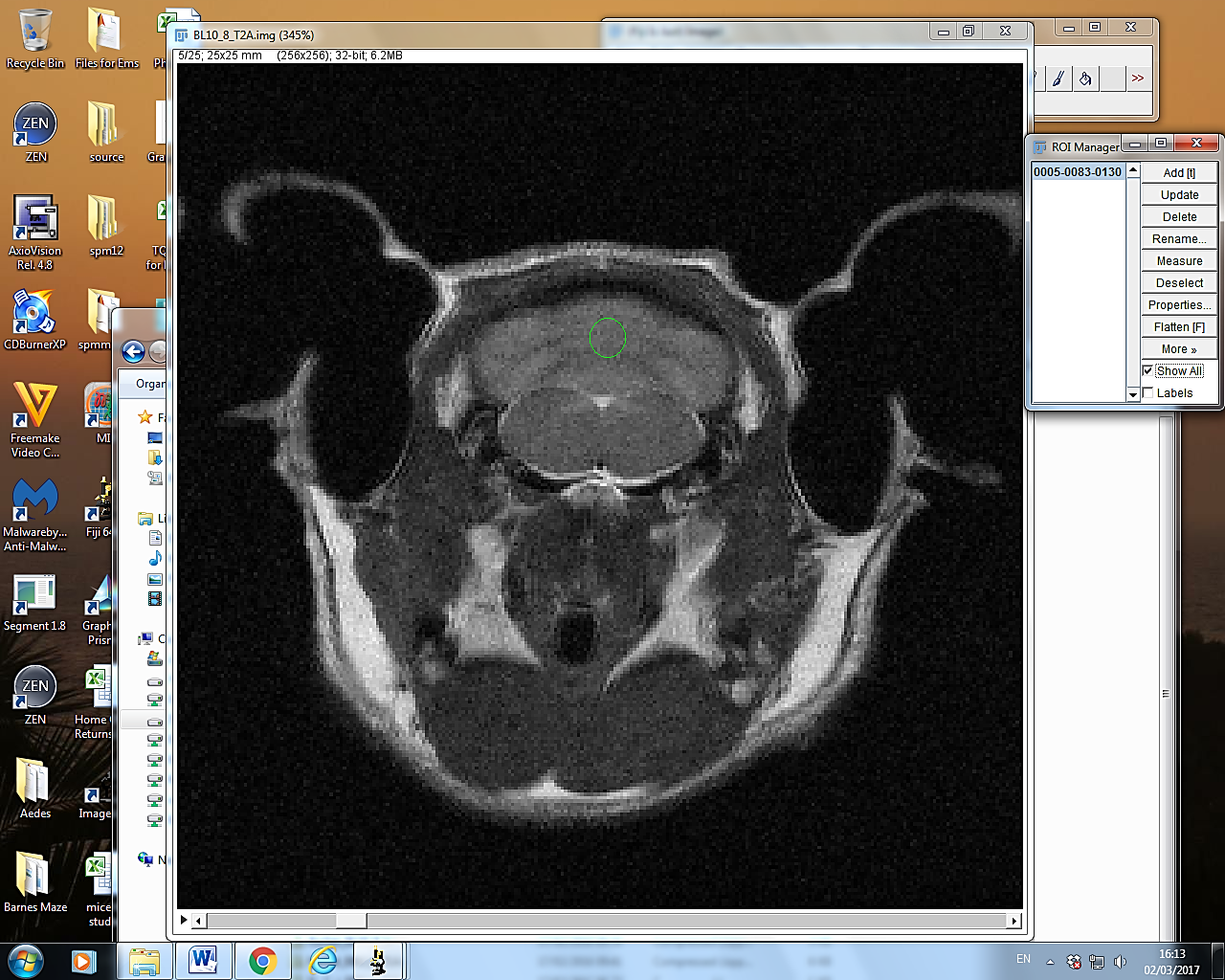


**ROI_5**

**e.**

**f.**

**ROI_4**

**Supplementary material 2**

Representative *T_2_-w* and *T_2_*-relaxometry MR images at 18 months old from *mdx* mice. a. Regions of interest (ROI), 1. hippocampus, 2. cerebral cortex and 3. corpus callosum, defined on a *T_2_*-weighted coronal image. b. Corresponding coronal *T_2_* map. c. ROI 4. caudate putamen defined on a *T_2_*-weighted coronal image d. Corresponding coronal *T_2_* map e. ROI 5 in the cerebellum defined on a *T_2_*-weighted coronal image. f. Corresponding coronal *T_2_* map.


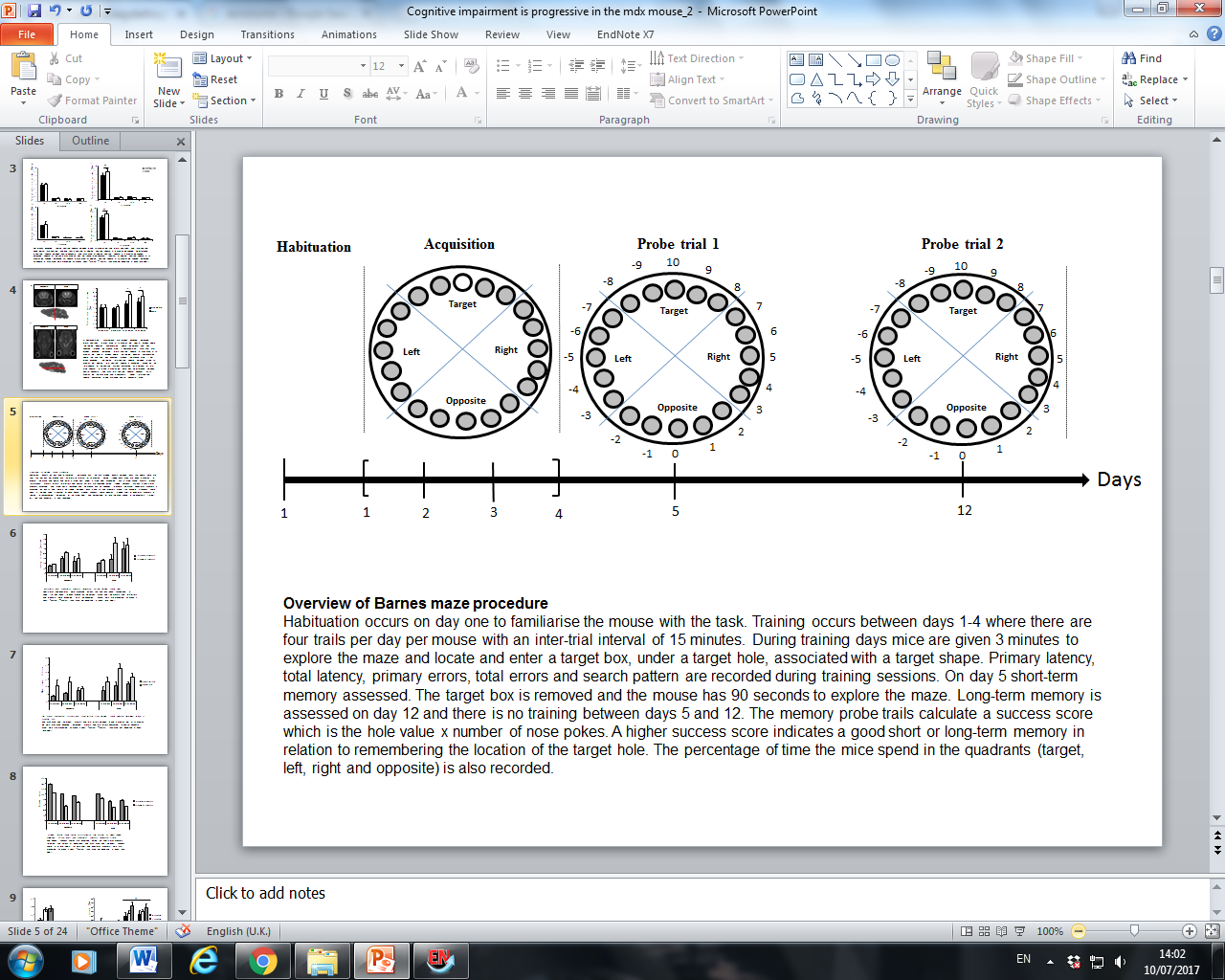


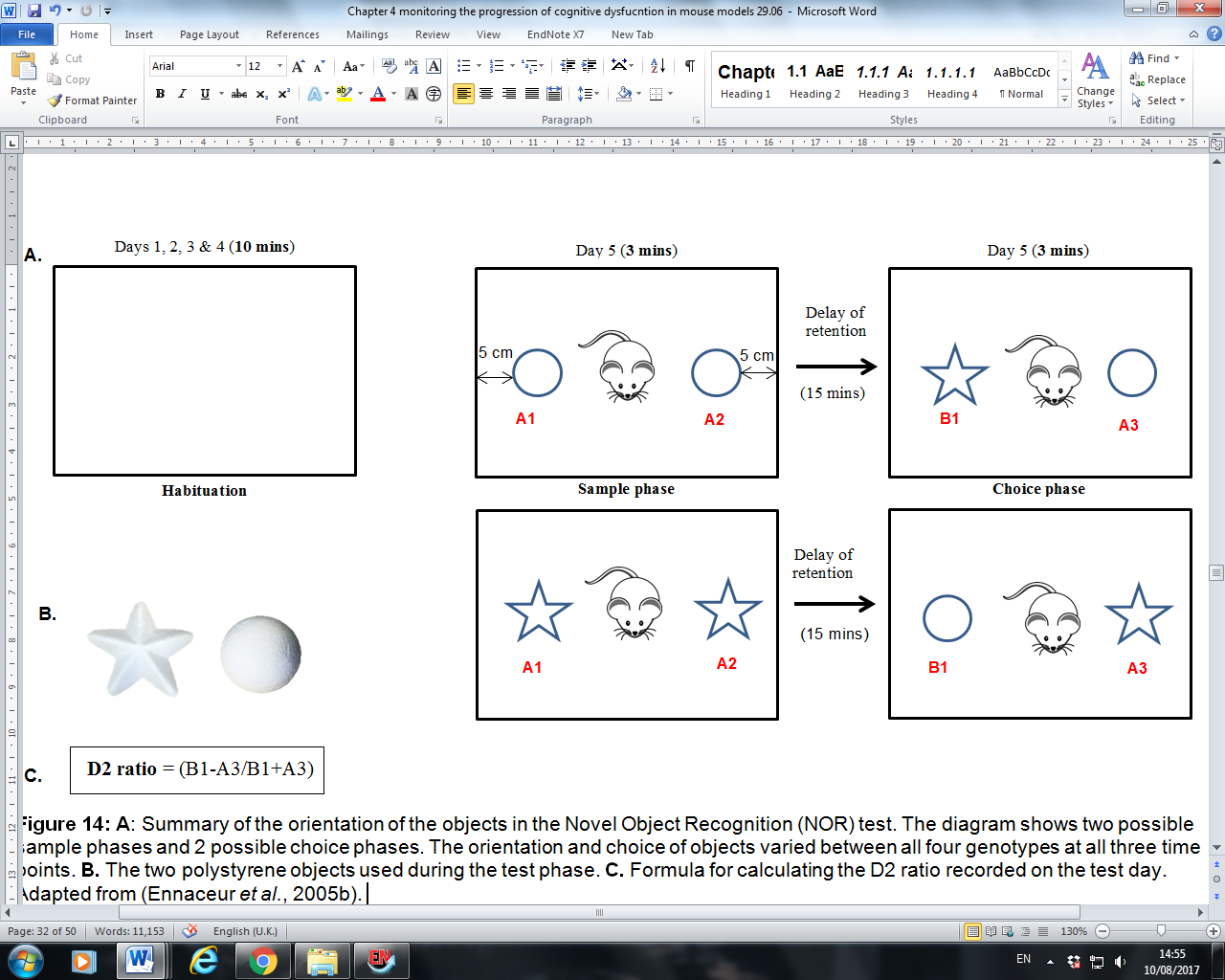


**a.**

**b.**

**Supplementary material 3**

**Behavioural testing overview.** **a.** Overview of the Barnes maze testing procedure. Habituation occurs on day 1 to familiarise the mouse with the task. Training occurs between days 1-4 where there are four trials per day per mouse with an inter-trial interval of 15 minutes. During training days mice are given 3 minutes to explore the maze and locate and enter a target box, under a target hole, associated with a target shape. Primary latency, total latency, primary errors, total errors and search pattern are recorded during training sessions. On day 5 short-term memory was assessed. The target box is removed, and the mouse has 90 seconds to explore the maze. Long-term memory is assessed on day 12 and there is no training between days 5 and 12. The memory probe trials calculate a success score which is the hole value x number of nose pokes. A higher success score indicates a good short or long-term memory in relation to remembering the location of the target hole. The percentage of time the mice spend in the quadrants (target, left, right and opposite) is also recorded. **b.** Summary of the orientation of the objects in the Novel Object Recognition (NOR) test. The diagram shows two possible sample phases and 2 possible choice phases. The orientation and choice of objects varied between both genotypes at all three time points. The two polystyrene objects used during the test phase and the formula for calculating the D2 ratio recorded on the test day (36)**.**

**The primary number of errors is the number of nose pokes into incorrect holes before the mouse reaches the target hole whereas the total number of errors is the number of errors committed before entering target hole throughout the whole trial. The *mdx* mice had the largest number of primary and total errors between 4 and 12 months old compared to control mice. The number of primary errors also increased with increasing age in the *mdx* mice. In the *mdx* mice the number of total errors increased between 4 and 6 months old but declined between 6 and 12 months old, this may be due to the increased freezing response exhibited by *mdx* mice at 12 months old. Control mice showed a slight increase in the number of primary errors between 4 and 12 months old but this was not seen with the number of total errors, in fact the number of total errors was reduced at 12 months old compared to 4 months old.**


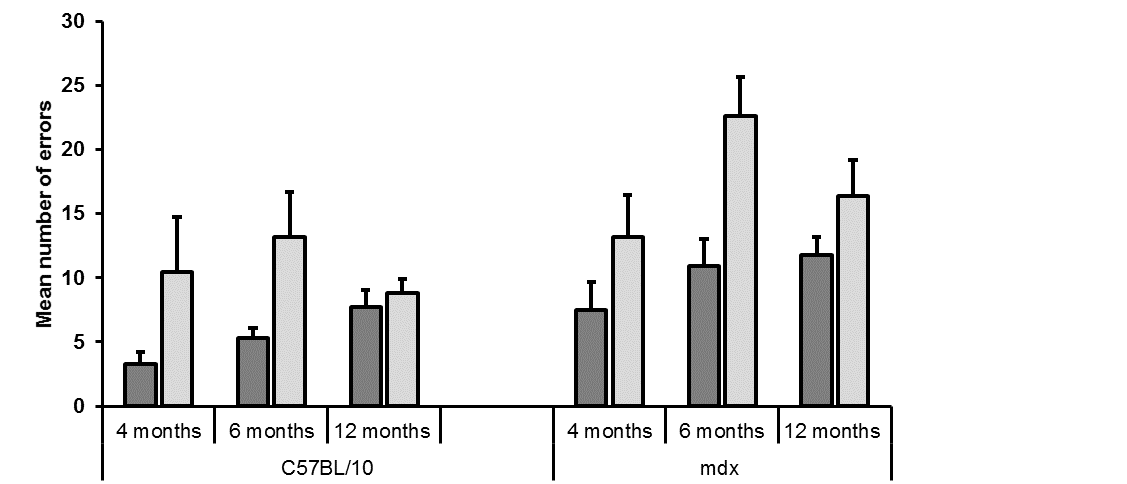


***C57BL/10***

***mdx***

**Mean number of errors**

**$**

****^^**


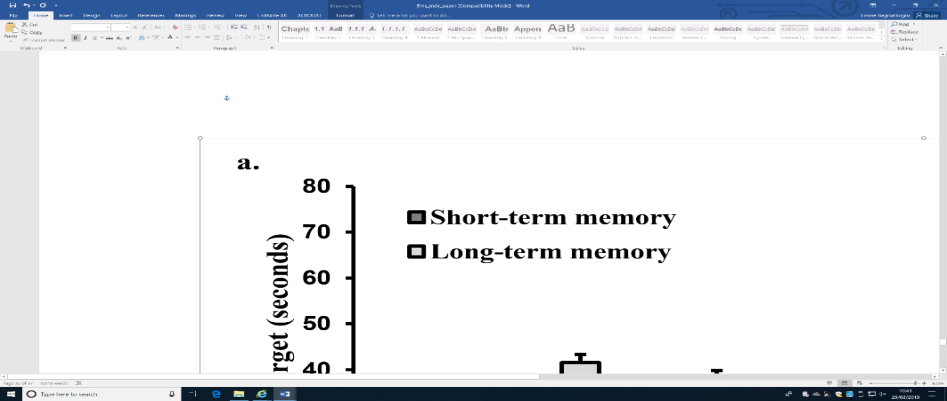


**Supplementary material 4**

**Bar graph displaying short- and long-term errors, from Barnes maze testing, between 4 and 12 months old.** The *mdx* mice had the largest amount of short- and long-term memory errors between 4 and 6 months old. At 12 months old the activity of *mdx* mice decreased due to increased anxiety-related behaviour, but the *mdx* mice still had higher amount of short- and long-term errors compared to control mice. At 4 months old the control mice had a higher mean number of errors for long-term memory compared to short-term memory (p<0.05), but this was not observed at 6 or 12 months old, nor was it higher than the mean number of errors recorded for the long-term memory of *mdx* mice at 4 months old. Whereas the *mdx* mice had an increased mean number of errors in long-term memory compared to short-term memory between 4 and 12 months old, with the 6 month old time point revealing the highest mean number of errors in long-term memory compared to both control mice (**p<0.01) and 4 month old mdx mice (^^p<0.01). Data presented as mean ±SEM, *p<0.05, **p<0.01 for control *vs mdx*, ^p<0.05, ^^p<0.01 for changes observed with ageing in *mdx* mice, $<p.0.05 for changes observed with ageing in control mice (n=8 mice per genotype/time point).

**4 months old**

**6 months old**


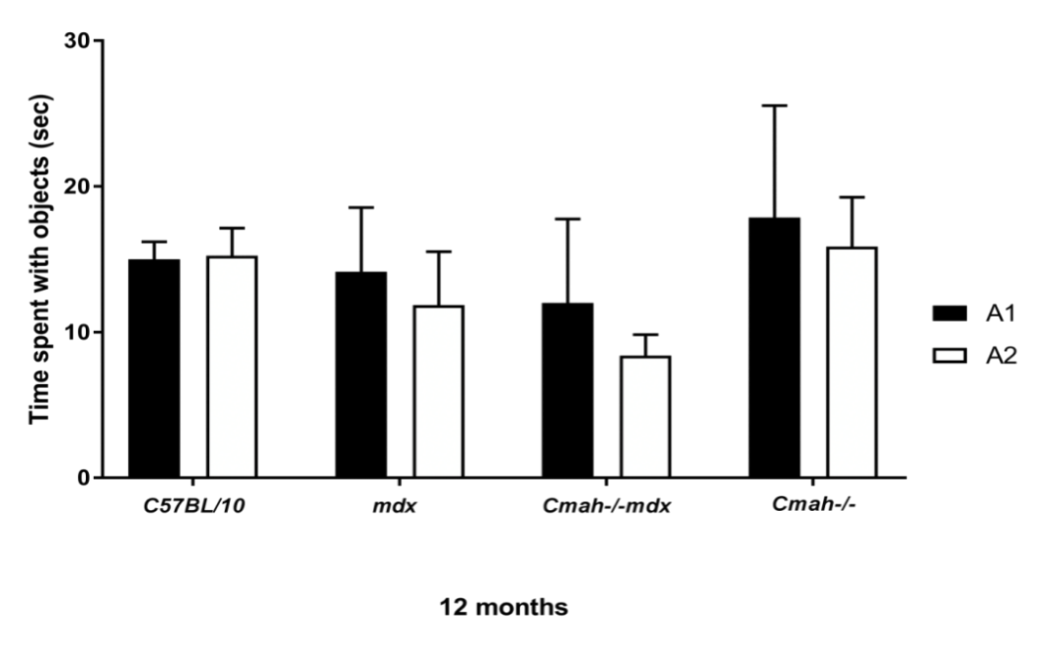

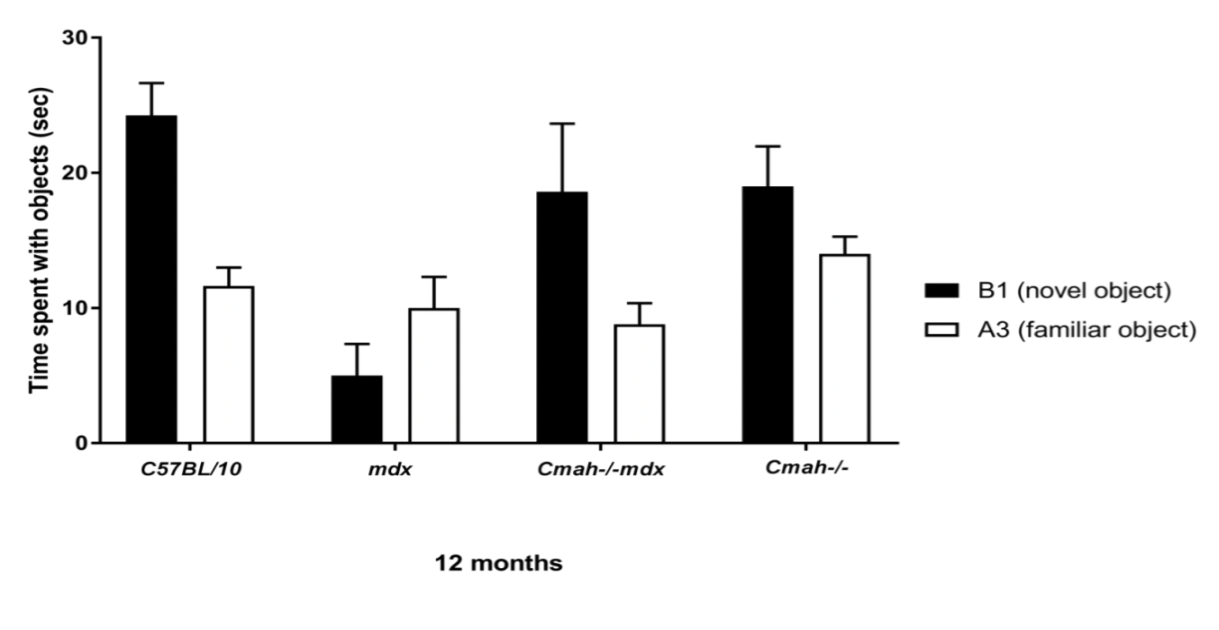


**a.**

**b.**

**c.**

**d.**

**e.**

**f.**

*

*

*

**

*

*

**12 months old**

**Supplementary material 5**

**Time spent with each object during the novel object recognition (NOR) task. a.** Time spent with objects in the sample phase of the NOR task at 4 months old. Graph shows that in all genotypes, mice showed no preference for either of the identical objects at 4 months old. **b.** Times spent with familiar and novel objects in the choice phase of the NOR task at 4 months old. Control mice showed a preference for the novel object whereas *mdx* mice showed a higher preference for the familiar object. **c.** Time spent with objects in the sample phase of the NOR task at 6 months old. Graph shows that in all genotypes, mice showed no preference for either of the identical objects at 6 months old. **d.** Times spent with familiar and novel objects in the choice phase of the NOR task at 6 months old. Control mice showed a preference for the novel object whereas *mdx* mice showed a higher preference for the familiar object. **e.** Time spent with objects in the sample phase of the NOR task at 12 months old. Graph shows that in all genotypes mice showed no preference for either of the identical objects at 12 months old. **f.** Times spent with familiar and novel objects in the choice phase of the NOR task at 12 months old. Control mice showed a preference for the novel object whereas *mdx* mice showed a higher preference for the familiar object. Data are presented as mean ± SEM, *p<0.05, **p<0.01, students t-test comparing preference for novel vs familiar object, ^p< 0.05, ^^p<0.01 (n=8 mice per genotype at each time point).
